# Metabolomics coupled with pathway analysis characterizes metabolic changes in response to BDE-3 induced reproductive toxicity in mice

**DOI:** 10.1038/s41598-018-23484-2

**Published:** 2018-04-03

**Authors:** Ziheng Wei, Jing Xi, Songyan Gao, Xinyue You, Na Li, Yiyi Cao, Liupeng Wang, Yang Luan, Xin Dong

**Affiliations:** 1 Faculty of Naval Medicine, Second Military Medical University, Shanghai, 200120 P. R. China; 2 Hongqiao International Institute of Medicine, Shanghai Tong Ren Hospital and Faculty of Public Health, Shanghai Jiao Tong University School of Medicine, Shanghai, 200025 P. R. China; 3 School of Pharmacy, Second Military Medical University, Shanghai, 200120 P. R. China

## Abstract

Polybrominated diphenyl ethers (PBDEs) may affect male reproductive function. 4-bromodiphenyl ether (BDE-3), the photodegradation products of higher brominated PBDEs, is the most fundamental mono-BDE in environment but is less studied. The purpose of this study was to investigate the reproductive toxicity induced by BDE-3 and explore the mechanism by metabolomics approach. In this study, mice were treated intragastrically with BDE-3 for consecutive six weeks at the dosages of 0.0015, 1.5, 10 and 30 mg/kg. The reproductive toxicity was evaluated by sperm analysis and histopathology examinations. UPLC-Q-TOF/MS was applied to profile the metabolites of testis tissue, urine and serum samples in the control and BDE-3 treated mice. Results showed the sperm count was dose-dependently decreased and percentage of abnormal sperms increased by the treatment of BDE-3. Histopathology examination also revealed changes in seminiferous tubules and epididymides in BDE-3 treated mice. Metabolomics analysis revealed that different BDE-3 groups showed metabolic disturbances to varying degrees. We identified 76, 38 and 31 differential metabolites in testis tissue, urine and serum respectively. Pathway analysis revealed several pathways including Tyrosine metabolism, Purine metabolism and Riboflavin metabolism, which may give a possible explanation for the toxic mechanism of BDE-3. This study indicates that UHPLC-Q-TOFMS-based metabolomics approach provided a better understanding of PBDEs-induced toxicity dynamically.

## Introduction

Polybrominated diphenyl ethers (PBDEs) are a class of brominated flame retardants that have emerged as a major environmental pollutant. In 2009, PBDEs were officially identified as a new class of persistent organic pollutants (POPs) by the United Nations Environment Programs (UNEP)^1^. Higher brominated diphenyl ethers can degrade in the environment to PBDEs with fewer bromines, the mono-BDE exhibits the longest photolysis lifetime among all the PBDEs in the air^2^. 4-bromodiphenyl ether (BDE-3) is the most fundamental mono-BDE in the environment and the most abundant photodegradation products of higher brominated PBDEs^3–6^. BDE-3 can be generated and accumulate in fish (Carassius auratus) by biotransformation of PBDEs^7^.

The health risk of PBDEs has recently raised global concern. PBDEs may disrupt the endocrine system, have neurodevelopment toxicity, teratogenicity and potential carcinogenicity^8,9^. PBDEs could affect the male reproductive function, exposure level of PBDEs in human serum is negatively associated with sperm mobility and concentration^10,11^. Exposure to 2,2′,4,4′-tetrabromodiphenyl ether (BDE-47) decreased the rate of sperm capacitation, altered sperm motility parameters and increased germ cell loss and apoptosis in both mice and rat^12,13^. Previous studies have shown that PBDEs with fewer bromines are more volatile and bioaccumulative^8,9^ and hence may be more harmful to human health^14^. Mono-BDE may induce genetic recombination in mammalian cells^15^. Our previous studies in *C*. *elegans* also demonstrated BDE-3 could induce reproductive dysfunction and germ cell apoptosis by induction of ROS and DNA damage^16^. By far, however, few study in mono-BDE toxicity have been reported in rodents^17^.

Metabolomics protocols are used to comprehensively characterize the metabolite content of biological samples by exploiting cutting-edge analytical platforms. Liquid chromatography-mass spectrometry technology platform is the most commonly used analytical technique for metabolomics research in recent years.

Nowadays, non-targeted metabolomics has gained widely attention as a method of profiling endogenous metabolites because of its high sensitivity, high throughput, and unbiasedness. Therefore, Metabolomics techniques were gradually applied to many aspects, such as evaluating and monitoring drugs and even atmospheric pollutant toxicity^18^. The analysis also showed enormous potential for the detection of health effect of POPs, e.g. metabolomic-based profiling revealed plasma responses to dioxin-associated dietary contaminant exposure^19^; identificated changed metabolites as potencial hepatotoxicity biomarker of Polychlorinated Biphenyls (PCBs) and 2,3,7,8-tetrachlorodibenzo-p-dioxin (TCDD)^20^; determinated toxic effects of PCBs and explained the mechanism of metabolic disturbance in obesity^21^; revealed the mechanisms of complex pesticide mixtures^22^ exposure increase oxidative stress and disturb energy metabolism.

In this study, we examined the effects of BDE-3 on the reproductive function in mice, with the lowest dose of 0.0015 mg/kg/day, which equal to the highest ingestion concentration of PBDEs in human (141 ng/kg/day) by the U.S. Environmental Protection Agency (EPA), 1.5 mg/kg/day, an equal dose to the lowest effective dose of its congener of BDE-47^17^, middle dose of 10 mg/kg/day and high dose of 30 mg/kg/day, respectively. We then performed metabolomics analyses for the testis, urine and serum samples after BDE-3 treatment using UPLC-Q-TOF/MS. The differential metabolites were identified by the pattern recognition using PLS-DA and univariate analysis for the control and BDE-3 treated mice. Our results may provide more evidence to better understand the mechanism of PBDEs-induced reproductive toxicity.

## Result

### Body, tissue weight and clinical observation

During the experimental period, animals were weighted once a week and no significant differences were observed in the body weight or clinical observation of the animals (Fig. S1 and Table S1). Testis and epididymis weight of each animal was measured on the day of the anatomy. No statistical differences in the testis weight or epididymis weight were found between the solvent group and BDE-3 groups after 6 weeks of BDE-3 treatment (Fig. S2).

### Sperm count, vitality and morphology

**Figure 1:**
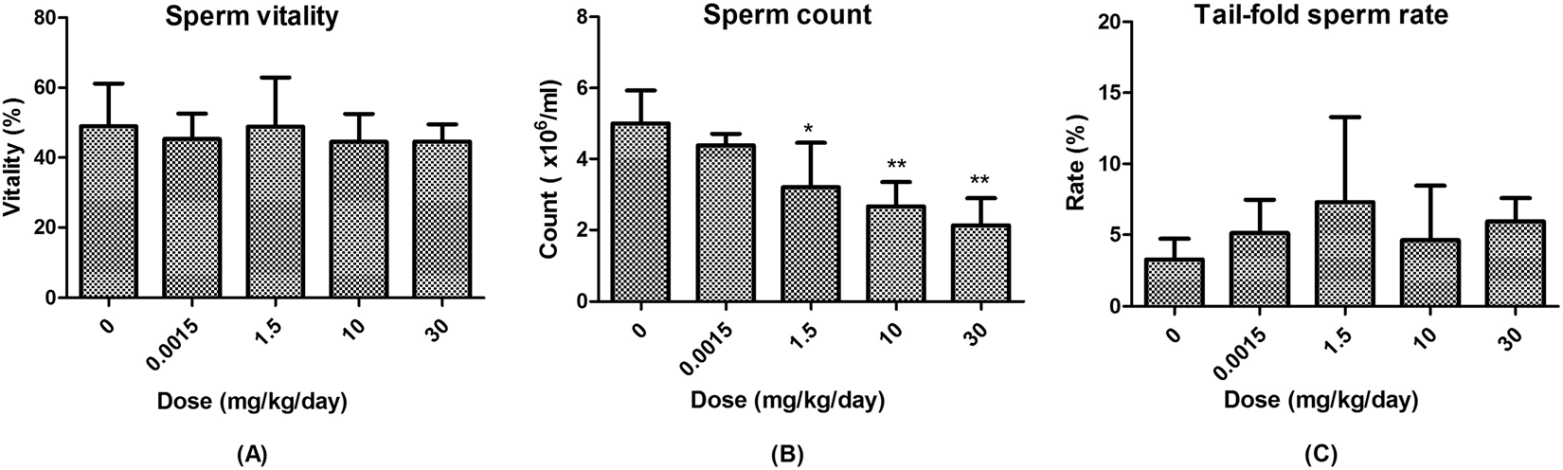
(**A**) Percentage of sperm vitality at mice treated with different concentration of BDE-3, mean ± SD (n = 6). (**B**) Count of sperm at mice treated with different concentration of BDE-3, mean ± SD (n = 6). *p < 0.05 versus solvent group, **p < 0.01 versus solvent group. (**C**) Percentage of tail-fold sperm at mice treated with different concentration of BDE-3, mean ± SD (n = 6).

BDE-3 treated mice showed no change in sperm vitality compare with vehicle control group, (Fig. 1A). However, the sperm count was dose-dependently decreased by the treatment of BDE-3 (Fig. 1B). The average counts of sperm at 1.5, 10 and 30 mg/kg dose groups were 3.2, 2.7, 2.1 × 106/mL, and were significantly decreased by 36%, 47% and 57% compared to the vehicle control group, respectively. Moreover, as shown in Fig. 1C, in the sperm morphology analysis, the rate of tail folding sperm in BDE-3 treated mice were increased 1.4 to 2.2-fold higher compared to the vehicle group although without statistical difference. No significant differences were observed in the banana gate, enlarged-headed, amorphous, double-headed and double-tailed (Fig. S3).

### Testis and epididymis histopathology

**Figure 2:**
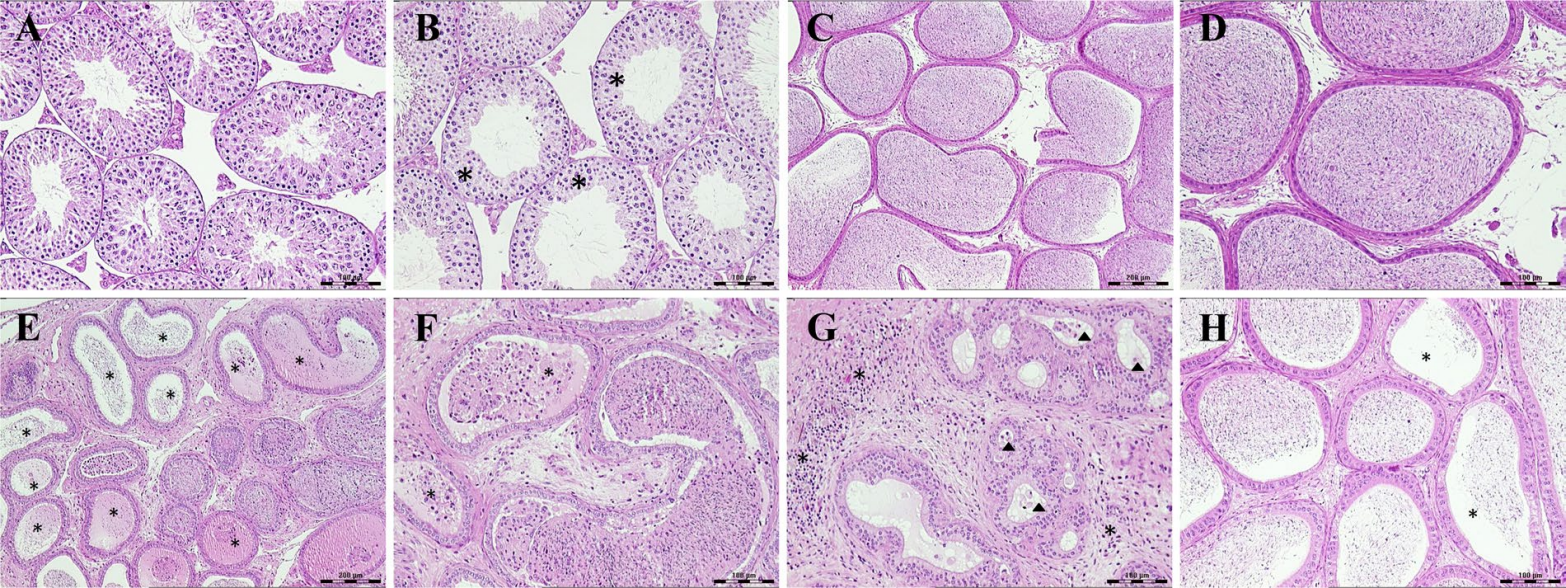
Photomicrographs of testes and epididymides. (**A**) Normal structure of seminiferous tubules from vehicle control group. (**B**) Mild germ cells decrease in seminiferous tubules from 30 mg/kg BDE-3 treated mice. (**C**,**D**) Normal structure of epididymides from vehicle control group. (**E**) Oligospermiain epididymal duct (*) from 30 mg/kg BDE-3 treated mice. (**F**) Cellular debris in epididymal duct (*) from 30 mg/kg BDE-3 treated mice. (**G**) Inflammatory cell infiltration in epididymal interstitium (*) and cellular debris in epididymal duct (∆) from 30 mg/kg BDE-3 treated mice. (**H**) Oligospermia in epididymal duct (*) from 30 mg/kg BDE-3 treated mice.

Compared with vehicle control group, minimal to mild germ cells decrease were found in seminiferous tubules in four males (4/6) of 30 mg/kg BDE-3 treated group as shown in Fig. 2A,B. Mature sperm decrease was revealed in epididymides in three males at 30 mg/kg BDE-3 treated group with 1 animal showed cellular debris in lumen and inflammatory cell infiltration in epididymal interstitium (Fig. 2C–H). No microscopically change was found in 0.0015, 1.5 and 10 mg/kg treated mice.

### Metabolomics profiling analysis

**Figure 3:**
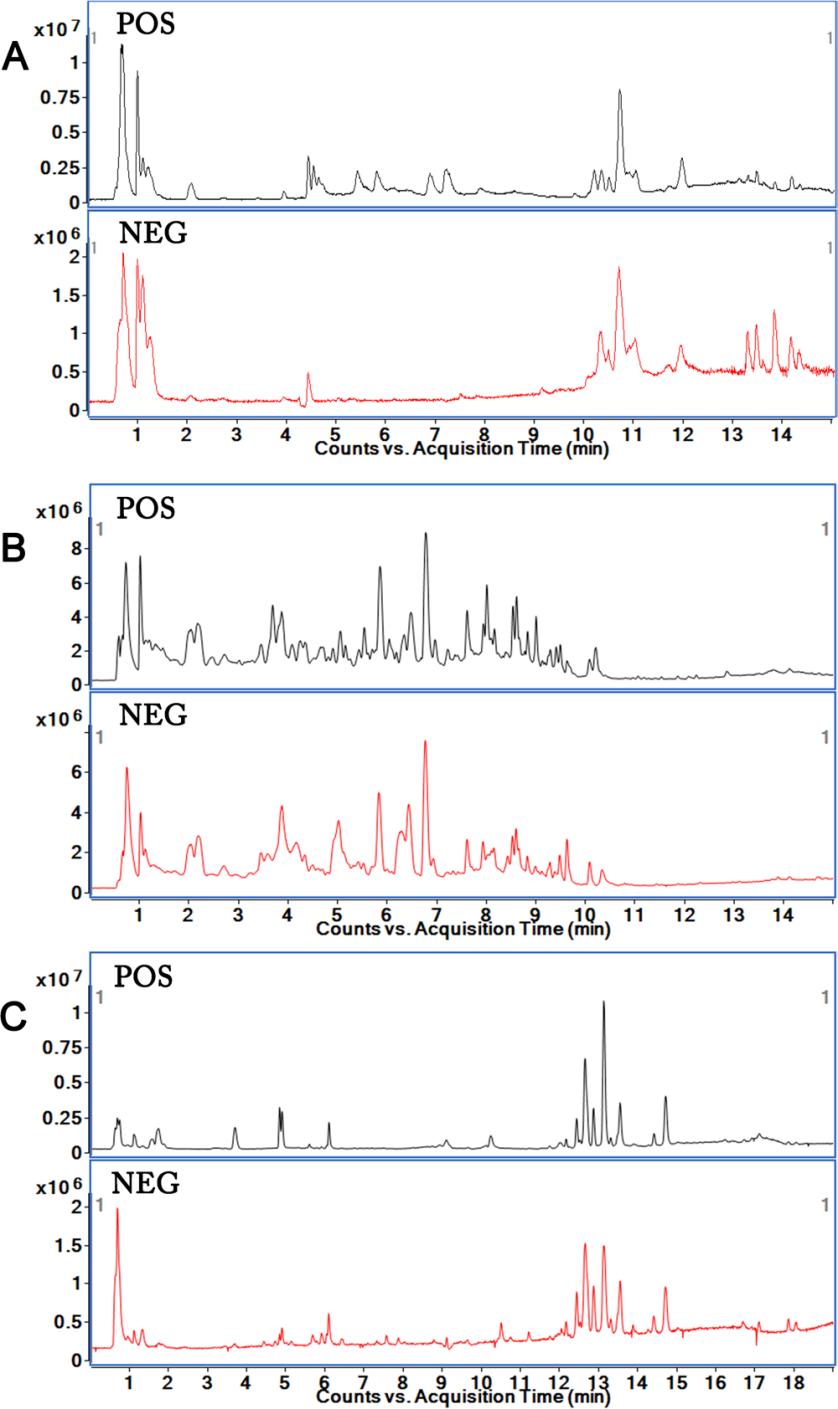
Representative total ion chromatograms (TICs) of samples based on UHPLC-Q-TOF/MS in electrospray ionization (ESI) positive and negative mode. (**A**) TICs of testis sample. (**B**) TICs of urine sample. (**C**) TICs of serum sample.

Metabolomic data of the testis, urine and serum samples were acquired using the abovementioned optimal conditions of UPLC-Q-TOF/MS. The representative total ion chromatograms (TICs) in both positive and negative ion modes of testis, urine and serum from the control and BDE-3 groups are shown in Fig. 3.

Initially, unsupervised PCA was used to observe the stability of the assay in the sequence between QC samples and other testis, urine and serum samples in different groups. Score plots from the PCA model have shown that the QC samples clustered together well in positive and negative mode, respectively, indicating the stability of the system was satisfying (Fig. S4).

In order to fully differentiate the testis, urine and serum metabolites between the control and the BDE-3 groups, the PLS-DA model was applied. PLS-DA is an efficient method to identify the ions that contribute to the clustering of samples. It also helps to eliminate non-correlated variations within the data set.

**Figure 4:**
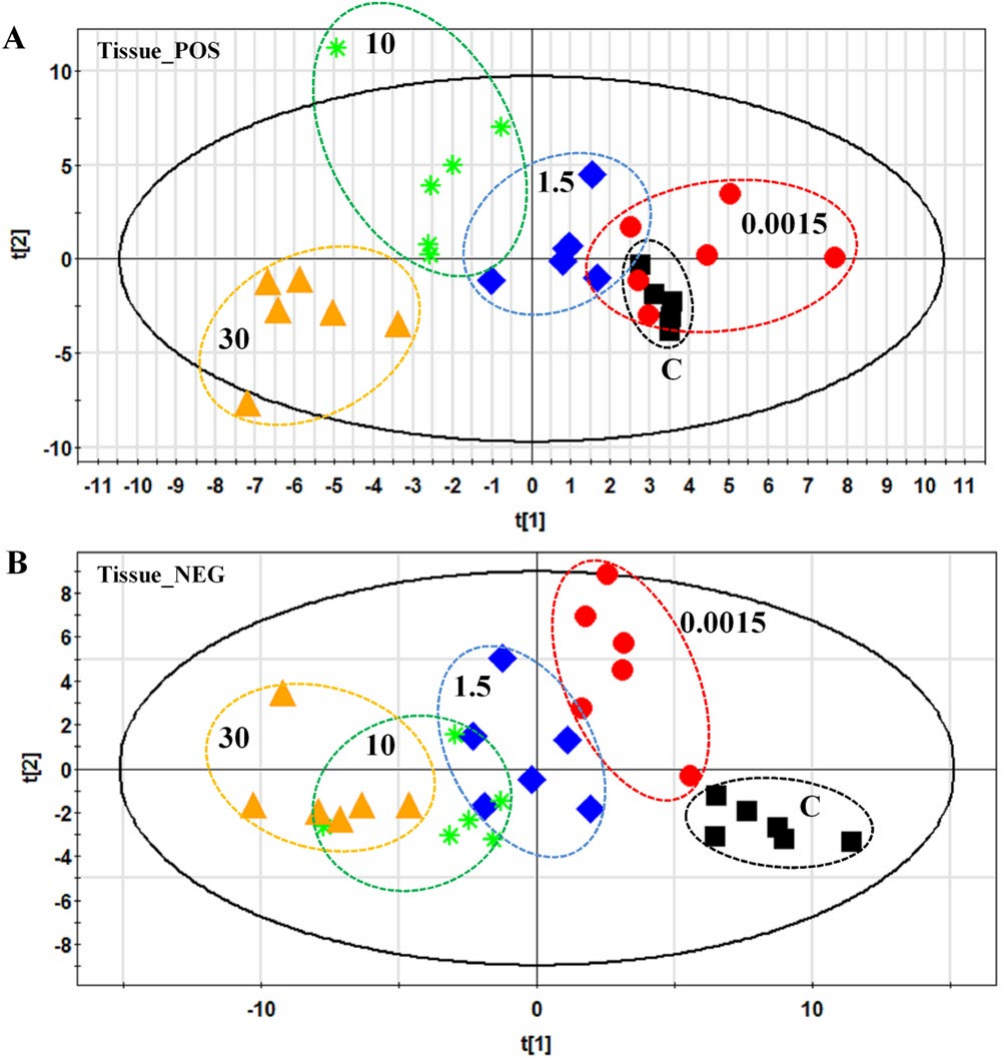
PLS-DA score plots of testis samples in the control group and BDE-3 groups at different dosages by RPLC-MS methods in positive (**A**) and negative (**B**) mode. (**A**) Testis samples of control group and all BDE-3 groups based on RP-MS methods in positive mode. (**B**) Testis samples of control group and all BDE-3 groups based on RP-MS methods in negative mode.

Score plots from the PLS-DA model have shown that the testicular samples from control group and dose groups clustered together respectively, and were clearly separated from each other (Fig. 4). Besides, the different BDE-3 groups exhibited an obvious separation trend, indicating that BDE-3 resulted in difference indeed among groups. The similar separation trend can also be observed in the score plots from the PLS-DA model of urine and serum samples (Figs S5 and S6).

In order to further differentiate the testicular metabolites, we applied PLS-DA model to characterize the differences between the control and each different BDE-3 groups. There was a distinct clustering between the control group and each BDE-3 groups at different dosages by UPLC-Q-TOF/MS method (Figs S7 and S8). The detailed model validation parameters (R2X, R2Y, and Q2) were shown on the Figs S7–S12. That showed good degree of fitting and predictive ability to screen the differential variables between groups.

The corresponding S-plot showed the contribution of different variables for the difference between the control and the BDE-3 groups. Each point in the S-plot represents an ion. Ions far away from the origin are significantly important to the differences between groups and have greater VIP values. Independent sample t-test was performed to assess the statistical significance. The important ions differentiating the BDE-3 groups from the control group at different dosages (VIP > 1, p < 0.05) were identified according our previous methods. Finally, 76 differentiated metabolites were identified in testis and listed in Table 1. Furthermore, we applied the PLS-DA model to characterize the differences among urine and serum samples in each group. Score plots from the PLS-DA model have shown that dose groups clustered together respectively, and were clearly separated from the control group (Figs S5 and S6). There was also a distinct separation of control group and each BDE-3 groups at different dosages by UPLC-Q-TOF/MS method (Figs S9–S12). We used T-Test method to screen the significantly changed metabolites between the control group and each BDE-3 groups at different dosages. Using the P < 0.05 and VIP > 1 as cut-off, 38 and 31 differentiated metabolites were identified in urine and serum respectively in positive mode and negative mode eventually (See Tables 2 and 3).

**Table 1:** Significantly differential metabolites in testicular tissue of BDE-3 treated mice.

No.	M/Z	RT(min)	Ion	VIP				HMDB	Metabolites	Formula	^a^FC(A)	FC(B)	FC(C)	FC(D)	Pathway
				0.0015	1.5	10	30								
1	184.073	0.717718	[M+H]+	1.08	2.03	2.57	0.53	HMDB0001565	Phosphocholine	C5H14NO4P	—	—	1.33^‡^	—	Sphingolipid metabolism
2	215.034	0.668722	[M+Cl]−	0.78	0.39	0.37	1.16	HMDB0000122	D-Glucose	C6H12O6	—	—	—	0.85*	Glycolysis
3	162.112	0.677051	[M+H]+	1.10	1.30	1.75	1.20	HMDB0000062	L-Carnitine	C7H15NO3	—	—	1.33*	—	Lysine degradation
4	90.055	0.698048	[M+H]+	0.31	0.85	1.56	0.19	HMDB0000161	Alanine	C3H7NO2	—	—	1.19^†^	—	Phenylalanine, tyrosine and tryptophan biosynthesis
5	132.077	0.700012	[M+H]+	4.13	9.42	11.54	1.57	HMDB0000064	Creatine	C4H9N3O2	—	—	1.22^‡^	—	Arginine and proline metabolism
6	116.07	0.712477	[M+H]+	2.00	1.15	1.45	1.39	HMDB0000162	L-Proline	C5H9NO2	1.14*	—	1.15*	—	Arginine and proline metabolism
7	346.047	0.729041	[M+H]+	0.13	1.55	1.28	1.01	HMDB0001314	cGMP	C10H12N5O7P	—	1.11*	1.15^†^	1.13^†^	cGMP-PKG signaling pathway
8	330.073	0.732617	[M+Na]+	0.74	1.35	1.00	0.91	HMDB0000125	Glutathione	C10H17N3O6S	1.08*	1.16^‡^	1.2^‡^	1.17^‡^	Glutathione metabolism
9	259.023	0.733917	[M−H]−	0.65	0.43	1.37	0.56	HMDB0001401	D-Glucose 6-phosphate	C6H13O9P	—	—	1.29^‡^	—	Glycolysis
10	171.007	0.7364	[M−H]−	0.30	0.98	1.55	0.86	—	D-Glycerol 1-phosphate	C3H9O6P	—	—	1.83^‡^	1.4^†^	
11	175.025	0.868175	[M−H]−	0.90	2.36	4.07	3.67	HMDB0000044	L-Ascorbic acid	C6H8O6	—	—	—	0.6*	
12	204.123	0.869535	[M+H]+	3.89	1.19	2.99	0.25	HMDB0000201	Acetylcarnitine	C9H17NO4	—	—	1.38*	—	Insulin resistance
13	565.048	0.87205	[M−H]−	1.21	0.88	1.67	1.18	HMDB0000286	UDP-glucose	C15H24N2O17P2	—	—	1.17^†^	1.15*	Pyrimidine metabolism
14	606.074	0.875378	[M−H]−	1.41	0.80	0.82	0.94	HMDB0000304	UDP-N-acetyl-D-galactosamine	C17H27N3O17P2	1.17*	—	—	1.19*	Amino sugar and nucleotide sugar metabolism
15	150.058	1.00794	[M+H]+	3.03	1.07	0.49	1.96	HMDB0000696	L-Methionine	C5H11NO2S	1.21^‡^	—	—	0.84^†^	Cysteine and methionine metabolism
16	137.046	1.01139	[M+H]+	2.26	3.94	2.61	4.59	HMDB0000157	Hypoxanthine	C5H4N4O	—	—	—	0.86^‡^	Purine metabolism
17	151.026	1.03714	[M−H]−	0.93	1.46	0.36	1.45	HMDB0000292	Xanthine	C5H4N4O2	—	0.88^†^	—	0.82^‡^	Purine metabolism
18	180.067	1.11158	[M−H]−	1.13	0.25	0.64	0.50	HMDB0000158	L-Tyrosine	C9H11NO3	1.22^†^	—	1.17*	—	Tyrosine metabolism
19	268.104	1.18454	[M+H]+	2.15	1.67	2.05	0.83	HMDB0000050	Adenosine	C10H13N5O4	0.79*	—	0.71^†^	—	Purine metabolism
20	132.102	1.20861	[M+H]+	9.50	4.85	0.75	3.67	HMDB0000687	L-Leucine	C6H13NO2	1.23^‡^	—	—	0.91*	Valine, leucine and isoleucine degradation
21	269.088	1.27399	[M+H]+	0.32	1.94	2.01	2.24	HMDB0000195	Inosine	C10H12N4O5	—	—	1.13^†^	1.13^†^	Purine metabolism
22	164.072	2.0733	[M−H]−	1.44	1.01	1.15	1.26	HMDB0000159	L-Phenylalanine	C9H11NO2	1.11*	—	0.88*	0.84^†^	Phenylalanine, tyrosine and tryptophan biosynthesis
23	357.089	2.45789	[M−H]−	0.45	0.45	0.98	1.37	HMDB0001416	D-Pantetheine 4′-phosphate	C11H23N2O7PS	—	—	1.12^†^	1.26^‡^	
24	220.118	2.69811	[M+H]+	0.58	0.22	0.89	1.11	HMDB0000210	Pantothenic Acid	C9H17NO5	—	—	0.92*	0.9^†^	Vitamin digestion and absorption
25	203.083	3.9441	[M−H]−	0.78	1.00	1.08	1.07	HMDB0000929	L-Tryptophan	C11H12N2O2	—	0.92*	0.87^†^	0.83^‡^	Tryptophan metabolism/Aminoacyl-tRNA biosynthesis/Phenylalanine, tyrosine and tryptophan biosynthesis
26	119.073	4.44353	[M+H]+	0.51	0.88	1.05	0.20	HMDB0040735	Ethyl lactate	C5H10O3	—	—	1.21^†^	—	Pyruvate metabolism
27	790.208	4.59486	[M+H]+	1.45	0.13	0.26	0.47	HMDB0001197	Reduced flavine adenine dinucleotide (FADH2)	C27H37N9O15P2	1.6^‡^	—	—	—	Riboflavin metabolism
28	677.484	4.59681	[M+H]+	1.34	0.19	0.28	0.56	HMDB0011204	PG(P-16:0/14:1)	C36H69O9P	1.52^†^	—	—	—	
29	131.072	5.05562	[M−H]−	0.57	1.05	0.88	0.85	HMDB0000665	Leucic acid	C6H12O3	0.88*	0.7^‡^	0.68^‡^	0.61^‡^	
30	779.581	5.82323	[M+H]+	0.26	1.09	1.29	2.26	HMDB0010602	PG(18:0/18:0)	C42H83O10P	—	—	1.3^‡^	0.24^‡^	
31	957.628	6.86155	[M+H]+	0.44	1.05	0.93	0.72	HMDB0009817	PI(21:0/22:4)	C52H93O13P	—	1.47^‡^	1.81^‡^	0.5^‡^	
32	157.061	7.51482	[M+Na]+	0.98	1.90	0.72	0.76	HMDB0012251	L-Canaline	C4H10N2O3	1.62	2.67^‡^	1.72	1.86	
33	621.303	10.0596	[M+H]+	1.44	1.86	1.43	1.80	HMDB0009897	PI(20:4/0:0)	C29H49O12P	0.81^‡^	0.73^‡^	0.66^‡^	0.46^‡^	
34	478.293	10.2657	[M+H]+	0.22	1.08	0.25	0.35	HMDB0011477	LysoPE(0:0/18:2)	C23H44NO7P	—	1.24^†^	—	—	
35	524.278	10.2884	[M−H]−	2.32	2.21	2.02	2.04	HMDB0011496	LysoPE(0:0/22:6)	C27H44NO7P	0.77^‡^	0.76^‡^	0.73^‡^	0.64^‡^	
36	1001.56	10.3001	[M+Cl]−	1.49	1.49	1.49	1.34	HMDB0009791	PI(22:2/22:4)	C53H91O13P	0.64^‡^	0.57^‡^	0.45^‡^	0.39^‡^	
37	520.34	10.304	[M+H]+	0.68	3.06	0.55	0.56	HMDB0010386	LysoPC(18:2)	C26H50NO7P	—	1.26^†^	—	—	
38	500.279	10.3084	[M−H]−	4.38	4.44	4.23	4.19	HMDB0011487	LysoPE(0:0/20:4)	C25H44NO7P	0.79^‡^	0.75^‡^	0.71^‡^	0.61^‡^	
39	568.34	10.3287	[M+H]+	3.44	1.46	2.39	1.93	HMDB0010404	LysoPC(22:6)	C30H50NO7P	0.83^†^	—	0.84^†^	0.87*	
40	1043.61	10.3316	[M+Cl]−	1.50	1.43	1.57	1.43	HMDB0004888	Ganglioside GA2 (d18:1/12:0)	C50H92N2O18	0.73^‡^	0.69^‡^	0.56^‡^	0.5^‡^	
41	566.321	10.3614	[M+Na]+	0.72	0.67	1.09	0.25	HMDB0010396	LysoPC(20:4)	C28H50NO7P	—	—	1.22^†^	—	
42	546.355	10.5766	[M+H]+	3.82	4.56	3.63	4.11	HMDB0010393	LysoPC(20:3)	C28H52NO7P	0.69^‡^	0.59^‡^	0.48^‡^	0.31^‡^	
43	448.342	10.5995	[M+H]+	1.07	0.88	1.02	1.04	HMDB0062343	N-stearoyl tyrosine	C27H45NO4	1.5*	—	1.73^‡^	1.76^‡^	
44	454.293	10.64	[M+H]+	1.82	4.81	4.33	5.75	HMDB0011473	LysoPE(0:0/16:0)	C21H44NO7P	—	0.91^†^	0.85^‡^	0.75^‡^	
45	571.289	10.6421	[M−H]−	1.03	1.27	0.64	1.53	—	PI(16:0/0:0)	C25H49O12P	—	—	—	0.89^†^	
46	496.268	10.6473	[M−H]−	1.31	1.07	1.01	0.93	—	PS(16:0/0:0)	C22H44NO9P	—	—	0.92*	—	
47	518.321	10.7014	[M+Na]+	1.73	2.28	2.22	1.66	HMDB0010382	LysoPC(16:0)	C24H50NO7P	—	1.19*	1.41^‡^	1.21*	
48	528.308	10.8622	[M+H]+	3.82	2.46	2.61	2.63	HMDB0011495	LysoPE(0:0/22:5)	C27H46NO7P	0.77^‡^	0.87*	0.8^‡^	0.77^‡^	
49	1095.64	10.8841	[M−H]−	1.53	1.33	1.32	1.07	HMDB0004842	Ganglioside GM3 (d18:1/12:0)	C53H96N2O21	0.52^‡^	0.55^‡^	0.43^‡^	0.48^‡^	
50	570.355	10.9047	[M+H]+	6.34	1.51	3.77	1.44	HMDB0010403	LysoPC(22:5)	C30H52NO7P	0.81^†^	0.95	0.85*	0.97	
51	438.299	10.9115	[M−H]−	1.51	0.97	1.70	0.50	HMDB0005780	PE(O-16:0/0:0)	C21H46NO6P	1.32^‡^	1.21*	1.63^‡^	0.86	
52	480.309	10.9772	[M+H]+	0.95	1.92	1.99	3.33	HMDB0011476	LysoPE(0:0/18:1)	C23H46NO7P	—	—	0.87^†^	0.74^‡^	
53	438.298	11.0137	[M+H]+	1.72	1.67	1.03	1.24	HMDB0011152	PE(P-16:0/0:0)	C21H44NO6P	1.11*	1.09*	1.09*	0.89*	
54	528.309	11.1544	[M−H]−	0.81	1.17	1.13	1.12	HMDB0011493	LysoPE(0:0/22:4)	C27H48NO7P	0.88^†^	0.76^‡^	0.69^‡^	0.59^‡^	
55	508.375	11.2894	[M+H]+	0.92	1.14	1.19	0.19	—	PC(O-18:1(9E)/0:0)	C26H54NO6P	—	1.24*	1.47^‡^	1.02	
56	483.273	11.6194	[M−H]−	1.55	1.47	1.92	1.04	—	PG(16:0/0:0)	C22H45O9P	0.92*	0.88^†^	0.76^‡^	0.89^†^	
57	556.339	11.6446	[M+Na]+	1.05	0.88	0.98	1.11	HMDB0011492	LysoPE(0:0/22:2)	C27H52NO7P	0.85^‡^	0.87^†^	0.74^‡^	0.67^‡^	
58	504.306	11.8836	[M+H]+	0.22	0.44	0.51	1.04	HMDB0011485	LysoPE(0:0/20:3)	C25H46NO7P	—	—	0.89*	0.65^‡^	
59	480.31	11.8854	[M−H]−	1.25	1.40	2.05	3.41	HMDB0011129	LysoPE(0:0/18:0)	C23H48NO7P	—	—	0.87^†^	0.61^‡^	
60	524.371	11.947	[M+H]+	5.50	4.13	4.66	8.38	HMDB0010384	LysoPC(18:0)	C26H54NO7P	1.08*	—	0.87^†^	0.71^‡^	
61	1047.73	11.9471	[M+Na]+	1.15	3.94	3.58	4.31	HMDB0055889	TG(22:5/22:6/22:6)	C69H100O6	—	0.76^†^	0.56^‡^	0.43^‡^	
62	546.353	11.9579	[M+H]+	1.10	1.33	1.30	2.27	—	PS(20:4/0:0)	C26H44NO9P	—	—	0.9*	0.78^‡^	
63	600.402	12.1688	[M+Na]+	0.23	1.10	1.25	0.95	HMDB0010399	LysoPC(22:1)	C30H60NO7P	—	—	0.75^‡^	0.85^†^	
64	524.299	12.2169	[M−H]−	0.45	1.14	1.07	1.56	—	PS(18:0/0:0)	C24H48NO9P	—	0.92*	0.88^†^	0.75^‡^	
65	466.329	12.2984	[M+H]+	0.18	0.23	0.19	1.02	—	PE(P-18:0/0:0)	C26H43NO6	—	—	—	0.63^‡^	
66	300.289	12.6925	[M+H]+	1.53	1.88	1.76	2.32	HMDB0000252	Sphingosine	C18H37NO2	0.8^‡^	0.74^‡^	0.53^‡^	0.7^‡^	Sphingolipid metabolism
67	756.551	12.9843	[M+H]+	0.59	1.87	2.42	1.41	HMDB0008166	PC(18:3/16:0)	C42H78NO8P	—	—	5.14^‡^	—	
68	303.233	13.4473	[M−H]−	3.87	4.80	4.13	4.31	HMDB0001043	Arachidonic Acid (peroxide free)	C20H32O2	0.89^‡^	0.83^‡^	0.82^‡^	0.75^‡^	Biosynthesis of unsaturated fatty acids
69	788.554	13.4499	[M+H]+	1.10	1.25	1.40	1.50	HMDB0012390	PS(18:1/18:1)	C42H78NO10P	0.68	0.67*	0.41^†^	0.34^‡^	
70	353.266	13.5693	[M+Na]+	0.54	1.12	1.01	0.83	—	1-Monopalmitin	C19H38O4	—	0.58^†^	0.31^‡^	0.47^‡^	
71	279.233	13.5874	[M−H]−	1.62	1.07	1.54	1.27	HMDB0000673	Linoleic acid	C18H32O2	0.76^†^	0.85*	0.68^‡^	0.68^‡^	Biosynthesis of unsaturated fatty acids
72	754.536	14.1461	[M+H]+	1.28	0.88	0.54	0.74	HMDB0007974	PC(16:0/18:4)	C42H76NO8P	0.79*	—	—	0.79*	
73	804.551	14.1466	[M+H]+	2.98	4.50	3.73	4.10	HMDB0008023	PC(16:1/22:6)	C46H78NO8P	0.84^†^	0.75^‡^	0.65^‡^	0.57^‡^	
74	805.554	14.1495	[M+H]+	1.62	1.28	1.40	2.42	HMDB0115266	PA(22:0/22:6)	C47H81O8P	—	—	—	0.81*	
75	255.233	14.1514	[M−H]−	2.04	1.39	1.42	1.39	HMDB0000220	Palmitic acid	C16H32O2	0.72^‡^	0.82^‡^	0.78^‡^	0.71^‡^	Biosynthesis of unsaturated fatty acids/Fatty acid metabolism
76	381.174	14.307	[M+Cl]-	1.23	1.98	2.07	2.02	HMDB0001547	corticosterone	C21H30O4	—	0.86^‡^	0.8^‡^	0.74^‡^	Steroid hormone biosynthesis

^a^FC, fold changes at different dosages, compared to the control mice. *p < 0.05, ^†^p < 0.01, ^‡^p < 0.001, “—” no statistically significant changes.

**Table 2:** Significantly differential metabolites in urine of BDE-3 treated mice.

NO.	M/Z	Ion	VIP				HMDB	Metabolite	Formula	^a^FC(A)	FC(B)	FC(C)	FC(D)	Pathway
			0.0015	1.5	10	30								
1	128.036	[M−H]−	0.77	0.60	1.42	0.89	HMDB0000267	Pyroglutamic acid	C5H7NO3	—	—	2.24^†^	—	Glutathione metabolism
2	129.021	[M−H]−	1.29	2.33	2.24	3.81	—	Acetylpyruvate	C5H6O4	—	—	—	0.62*	
3	130.052	[M−H]−	0.59	0.23	1.30	0.22	HMDB0002113	3-Hydroxy-L-proline	C5H9NO3	—	—	—	0.78*	
4	132.077	[M+H]+	0.96	0.96	3.79	2.62	HMDB0000064	Creatine	C4H9N3O2	—	0.33*	—	—	Arginine and proline metabolism
5	132.101	[M+H]+	2.22	2.22	2.09	1.45	HMDB0000687	L-Leucine	C6H13NO2	0.79*	0.73*	—	—	Valine, leucine and isoleucine degradation
6	136.039	[M+H]+	1.99	1.99	1.28	1.32	HMDB0000742	L-Homocysteine	C4H9NO2S	1.88^†^	2.24^†^	—	—	Biosynthesis of amino acids
7	137.071	[M+Na]+	0.31	0.31	1.60	1.28	—	L-Prolinamide	C5H10N2O	—	2.08*	—	—	
8	139.051	[M+H]+	1.07	1.07	1.71	1.77	HMDB0002730	Nicotinamide N-oxide	C6H6N2O2	—	—	1.73^†^	—	
9	145.016	[M+FA−H]−	0.40	1.21	0.69	2.32	HMDB0032523	Succinic anhydride	C4H4O3	0.78*	—	—	—	
10	162.079	[M+H]+	1.11	1.11	2.65	1.74	—	Alanopine	C6H11NO4	0.68^‡^	0.51^‡^	—	—	
11	170.043	[M+Na]+	0.94	0.94	0.96	0.59	—	L-Glutamate	C5H9NO4	1.61*	1.83^†^	—	—	D-Glutamine and D-glutamate metabolism
12	171.067	[M+Na]+	1.19	1.19	1.03	0.59	HMDB0000227	Mevalonic acid	C6H12O4	—	—	—	1.35*	Pantothenate and CoA biosynthesis
13	176.039	[M−H]−	1.32	0.99	2.39	1.54	HMDB0001015	N-Formylmethionine	C6H11NO3S	—	—	1.61*	—	Cysteine and methionine metabolism
14	189.098	[M−H]−	1.45	1.43	0.26	0.38	—	beta-Hydroxyarginine	C6H14N4O3	—	—	—	0.65*	Arginine and proline metabolism
15	190.107	[M+H]+	0.94	0.94	0.48	0.70	—	Alexine	C8H15NO4	—	—	1.72*	1.89^†^	
16	204.069	[M+Na]+	1.57	1.57	0.89	0.65	HMDB0000158	L-Tyrosine	C9H11NO3	—	0.51*	—	—	Tyrosine metabolism
17	206.044	[M+H]+	3.33	3.33	5.59	4.68	HMDB0000881	Xanthurenic acid	C10H7NO4	—	—	0.15*	—	Tryptophan metabolism
18	226.143	[M+H]+	1.23	1.23	1.62	0.80	—	Prenalterol	C12H19NO3	—	—	—	1.45*	
19	232.117	[M+H]+	1.68	1.68	2.86	3.68	HMDB0000953	Suberylglycine	C10H17NO5	—	—	—	1.29*	
20	238.022	[M+FA−H]−	0.54	0.56	1.29	1.68	HMDB0041624	Phosphocreatinine	C4H8N3O4P	—	—	1.37*	1.45*	
21	241.093	[M+Na]+	0.73	0.73	0.99	1.19	HMDB0001238	N-Acetylserotonin	C12H14N2O2	—	—	1.81^‡^	—	Tryptophan metabolism
22	245.011	[M−H]−	2.68	1.28	0.86	4.32	—	Phosphatidyl glycerol	C6H15O8P	—	—	2.97^‡^	3.45^†^	Glycerophospholipid metabolism
23	245.095	[M+H]+	0.44	0.44	0.88	1.89	HMDB0000030	D-Biotin	C10H16N2O3S	0.66*	—	—	—	Biotin metabolism
24	248.113	[M+H]+	1.60	1.60	2.00	1.89	HMDB0002095	Malonylcarnitine	C10H17NO6	—	—	1.38*	1.39*	
25	258.104	[M+H]+	1.18	1.18	0.67	0.60	HMDB0000982	5-Methylcytidine	C10H15N3O5	—	—	1.34*	—	
26	268.104	[M+H]+	2.81	2.81	2.53	0.57	HMDB0000050	Adenosine	C10H13N5O4	—	—	1.30*	—	Purine metabolism
27	274.11	[M+H]+	0.32	0.32	0.33	0.71	HMDB0000667	L-Thyronine	C15H15NO4	—	0.42^†^	—	—	
28	283.114	[M+H]+	0.23	0.23	1.03	1.14	—	2-Aminoadenosine	C10H14N6O4	—	—	1.28*	—	
29	319.184	[M+H]+	1.07	1.07	1.46	1.28	HMDB0006709	Ubiquinone (Q2)	C19H26O4	—	—	1.76*	—	Oxidative phosphorylation
30	321.044	[M−H]−	4.12	3.84	1.42	1.64	—	dTMP	C10H15N2O8P	1.33*	1.36*	—	—	Pyrimidine metabolism
31	348.088	[M−H]−	1.13	1.09	0.16	0.91	HMDB0060507	S-(Formylmethyl)glutathione	C12H19N3O7S	—	1.59^†^	1.80^†^	1.91^†^	Metabolism of xenobiotics by cytochrome P450
32	375.131	[M−H]−	1.18	1.30	0.56	0.69	HMDB0000244	Riboflavin (Vitamin B2)	C17H20N4O6	—	—	—	0.54*	Riboflavin metabolism
33	377.145	[M−H]−	1.32	1.36	0.82	0.84	HMDB0001577	Reduced riboflavin	C17H22N4O6	0.87*	—	0.84*	—	Riboflavin metabolism
34	397.235	[M+FA-H]-	2.84	2.52	0.42	0.90	HMDB0001220	PGE2	C20H32O5	—	—	0.63*	—	Arachidonic acid metabolism
35	482.361	[M+H]+	0.11	0.11	1.21	0.91	—	Lyso-PAF C-16	C24H52NO6P	—	—	0.68*	—	Ether lipid metabolism
36	496.34	[M+H]+	1.22	1.22	2.04	0.97	HMDB0010382	LysoPC(16:0)	C24H50NO7P	—	—	—	1.20*	Glycerophospholipid metabolism
37	569.333	[M+FA-H]-	0.91	1.82	0.68	0.43	—	PG(P-20:0/0:0)	C26H53O8P	—	0.75*	0.58^‡^	0.72^†^	
38	605.309	[M+FA-H]-	0.62	1.44	0.48	0.32	—	PG(22:4/0:0)	C28H49O9P	0.80*	0.61^†^	0.77*	—	

^a^FC, fold changes at different dosages, compared to the control mice. *p < 0.05, ^†^p < 0.01, ^‡^p < 0.001, “—” no statistically significant changes.

**Table 3:** Significantly differential metabolites in serum of BDE-3 treated mice.

NO.	m/z	Ion	VIP				HMDB	Metabolite	Formula	^a^FC(0.0015)	FC(1.5)	FC(10)	FC(30)	Pathway
			0.0015	1.5	10	30								
1	116.071	[M+H]+	1.85	1.62	1.34	1.37	HMDB0000162	L-Proline	C5H9NO2	1.46^*^	1.65^†^	—	1.51^*^	Arginine and proline metabolism
2	118.086	[M+H]+	2.43	2.58	1.93	2.05	HMDB0000883	L-Valine	C5H11NO2	—	1.51^†^	1.41^*^	1.47^*^	Valine, leucine and isoleucine degradation
3	129.056	[M−H]−	0.81	1.38	1.46	1.86	HMDB0000695	Ketoleucine	C6H10O3	—	1.71^*^	1.57^*^	1.82^†^	Valine, leucine and isoleucine degradation
4	130.087	[M−H]−	0.96	1.13	1.08	0.99	HMDB0000687	L-Leucine	C6H13NO2	—	1.58^†^	1.46^†^	1.39^*^	Valine, leucine and isoleucine degradation
5	136.076	[M+H]+	1.50	0.84	1.41	0.85	HMDB0010715	2-Phenylacetamide	C8H9NO	1.42^†^	1.35^*^	1.76^†^	1.33^*^	Phenylalanine metabolism
6	150.058	[M+H]+	2.17	1.44	1.42	1.17	HMDB0000696	L-Methionine	C5H11NO2S	1.34^*^	1.33^*^	—	—	Cysteine and methionine metabolism
7	157.061	[M+H]+	1.28	0.89	0.56	1.22	HMDB0001014	4-Imidazolone-5-propionic acid	C6H8N2O3	1.53^‡^	1.50^*^	1.56^†^	1.45^†^	Histidine metabolism
8	162.112	[M+H]+	1.55	0.61	1.01	1.57	HMDB0000062	L-Carnitine	C7H15NO3	—	—	—	1.34^*^	Lysine degradation
9	164.071	[M−H]−	1.18	1.29	1.24	1.01	HMDB0000159	L-Phenylalanine	C9H11NO2	1.26^*^	1.36^†^	1.34^†^	1.25^*^	Phenylalanine, tyrosine and tryptophan biosynthesis
10	165.055	[M+H]+	1.44	0.83	1.41	0.82	—	L-Tyrosine fragment	C9H8O3	1.40^†^	1.34^*^	1.77^†^	1.31^*^	
11	172.991	[M−H]−	1.89	1.27	0.24	1.38	—	Phenyl sulfate	C6H6O4S	2.61^*^	1.73^*^	—	2.11^*^	
12	182.081	[M+H]+	3.61	1.93	3.34	1.94	HMDB0000158	L-Tyrosine	C9H11NO3	1.42^†^	1.33^*^	1.75^†^	1.31^*^	Tyrosine metabolism
13	185.154	[M+H]+	1.45	1.34	1.39	1.50	—	2-hendecenoic acid	C11H20O2	1.92^‡^	2.28^‡^	2.29^‡^	2.44^‡^	
14	188.071	[M+H]+	3.30	1.57	2.80	2.31	—	L-Tryptophan fragment	C11H9NO2	1.25^†^	—	1.32^‡^	1.24^†^	
15	188.986	[M−H]−	1.21	1.35	0.32	1.16	HMDB0059724	Pyrocatechol sulfate	C6H6O5S	1.87^*^	2.10^*^	—	1.91^*^	
16	203.082	[M−H]−	1.68	0.74	1.46	1.05	HMDB0000929	L-Tryptophan	C11H12N2O2	1.19^†^	—	1.20^†^	1.12^*^	Phenylalanine, tyrosine and tryptophan biosynthesis
17	212.002	[M−H]−	2.74	3.14	1.23	2.28	—	Indoxylsulfuric acid	C8H7NO4S	2.08^†^	2.22^†^	—	1.95^*^	
18	225.123	[M+NH4]+	1.62	1.13	0.68	1.56	HMDB0000512	N-Acetyl-L-phenylalanine	C11H13NO3	0.50^†^	0.43^†^	—	0.35^‡^	Phenylalanine metabolism
19	231.159	[M+H]+	1.22	1.11	1.11	1.23	HMDB0000623	Dodecanedioic acid	C12H22O4	1.95^‡^	2.30^‡^	2.24^‡^	2.46^‡^	
20	267.073	[M−H]−	0.73	0.44	2.18	0.58	HMDB0000195	Inosine	C10H12N4O5	—	—	3.26^‡^	—	Purine metabolism
21	454.294	[M+H]+	1.40	1.19	1.10	1.04	HMDB0011473	LysoPE(0:0/16:0)	C21H44NO7P	1.27^*^	1.44^†^	1.32^*^	1.30^*^	
22	478.293	[M−H]−	0.63	1.31	1.10	0.72	HMDB0011505	LysoPE(18:1/0:0)	C23H46NO7P	—	1.73^†^	1.43^†^	—	Pentose and glucuronate interconversions
23	478.294	[M+H]+	0.16	2.91	2.18	0.58	HMDB0011507	LysoPE(18:2/0:0)	C23H44NO7P	—	1.92^‡^	1.51^†^	—	
24	494.325	[M+H]+	4.34	2.99	3.84	2.74	HMDB0010383	LysoPC(16:1)	C24H48NO7P	1.81^‡^	2.06^*^	2.14^‡^	1.69^†^	Glycerophospholipid metabolism
25	518.323	[M+H]+	1.59	1.53	1.56	1.47	HMDB0010387	LysoPC(18:3)	C26H48NO7P	1.09^*^	1.16^‡^	1.15^‡^	1.14^†^	Pentose and glucuronate interconversions
26	522.357	[M+H]+	8.72	7.06	8.03	6.31	HMDB0010385	LysoPC(18:1)	C26H52NO7P	1.49^†^	1.74^†^	1.76^†^	1.52^*^	
27	542.325	[M+H]+	2.14	2.06	2.51	1.87	HMDB0010397	LysoPC(20:5/0:0)	C28H48NO7P	1.44^‡^	1.94^†^	1.97^‡^	1.63^‡^	
28	544.341	[M+H]+	0.81	1.71	2.55	1.20	HMDB0010396	LysoPC(20:4)	C28H50NO7P	—	—	1.20^*^	—	Glycerophospholipid metabolism
29	546.357	[M+H]+	2.17	1.86	3.03	2.53	HMDB0010393	LysoPC(20:3)	C28H52NO7P	—	1.45^*^	1.80^†^	1.53^†^	Pentose and glucuronate interconversions
30	570.356	[M+H]+	1.14	1.05	1.39	1.04	HMDB0010403	LysoPC(22:5)	C30H52NO7P	—	2.01^†^	2.03^†^	—	Pentose and glucuronate interconversions
31	619.288	[M+Cl]-	0.37	1.64	1.76	0.81	HMDB0002577	Cholic acid glucuronide	C30H48O11	—	1.89^†^	1.84^†^	—	Pentose and glucuronate interconversions

^a^FC, fold changes at different dosages, compared to the control mice. *p < 0.05, ^†^p < 0.01, ^‡^p < 0.001, “—” no statistically significant changes.

**Figure 5:**
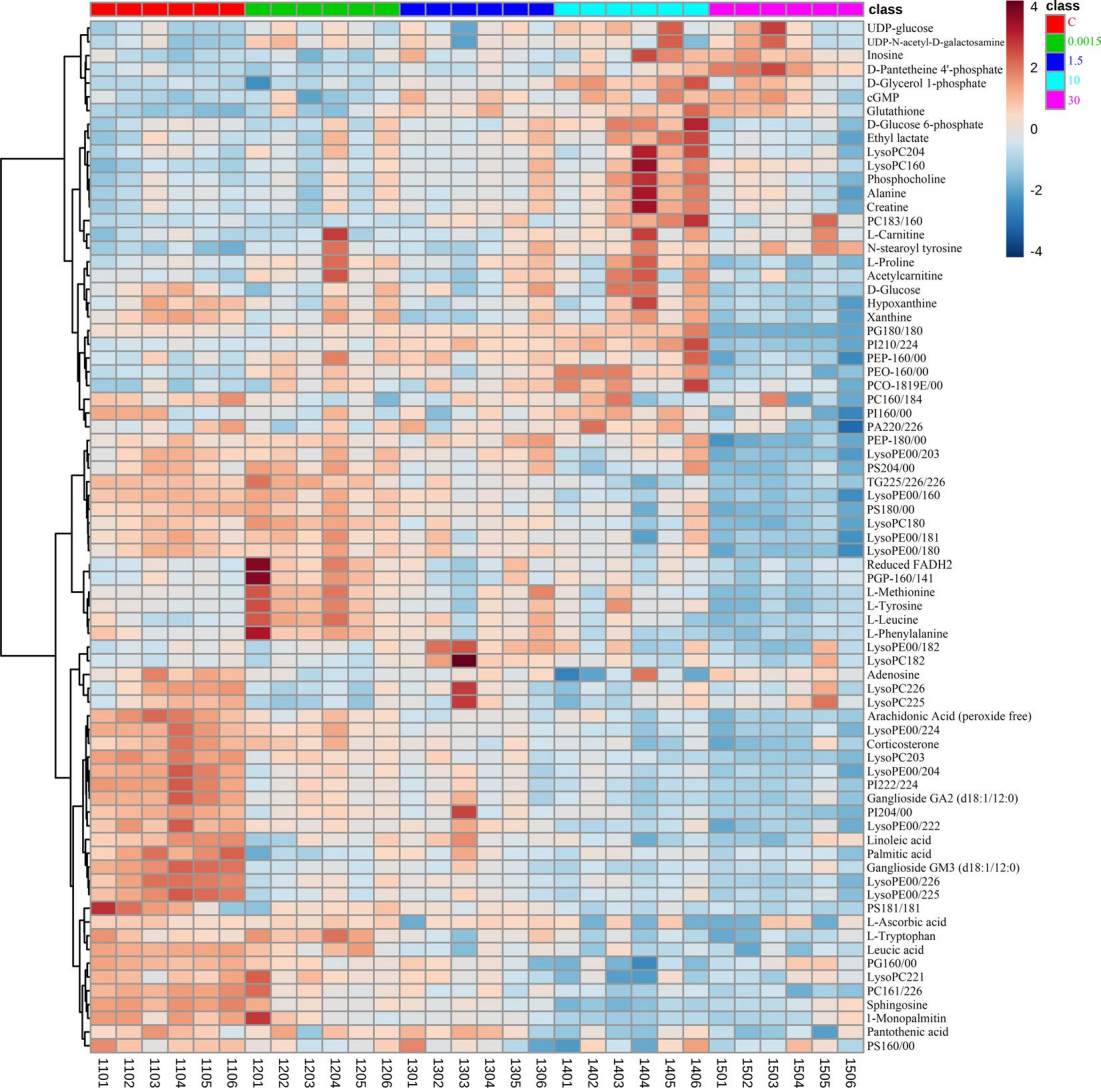
The clustering heat map of the control (C) and BDE-3 mice across different dosages (0.0015, 1.5, 10, 30) based on the 76 differentially metabolites in testicular tissue. Each column is labeled with different colors according to the sample type.

Heat maps can visually display the gradient changes of the differential metabolites between the control and BDE-3 groups. The heat map was constructed (Fig. 5) based on the normalized data set of the differential metabolites in testis, urine and serum. According to our results, in testicular tissue, Phosphocholine, L-Carnitine, Alanine, Creatine, L-Proline, cGMP, Glutathione, D-Glucose 6-phosphate, D-Glycerol 1-phosphate, Acetylcarnitine, UDP-glucose, UDP-N-acetyl-D-galactosamine, L-Tyrosine, Inosine, D-Pantetheine 4′-phosphate, Reduced flavine adenine dinucleotide (FADH2), N-stearoyl tyrosine and PG(P-16:0/14:1) were increased in BDE-3 treated groups. Leucic acid, Ganglioside GA2 (d18:1/12:0), Ganglioside GM3 (d18:1/12:0), Arachidonic Acid (peroxide free), Palmitic acid, corticosterone and amount of lipids metabolites were obviously decreased in the BDE-3 groups compared with the control group.

In urine (Fig. S13A), 2-Aminoadenosine, 5-Methylcytidine, Adenosine, Alexine, dTMP, L-Glutamate, L-Homocysteine, L-Prolinamide, LysoPC(16:0), Malonylcarnitine, Mevalonic acid, N-Acetylserotonin, N-Formylmethionine, Nicotinamide N-oxide, Phosphatidyl glycerol, Phosphocreatinine, Prenalterol, Pyroglutamic acid, S-(Formylmethyl)glutathione, Suberylglycine, Ubiquinone (Q2)significantly increased in the BDE-3 groups compared with the control group. However, the metabolites including 3-Hydroxy-L-proline, Acetylpyruvate, Alanopine, beta-Hydroxyarginine, Creatine, D-Biotin, L-Leucine, L-Thyronine, L-Tyrosine, Lyso-PAF C-16, PG(22:4/0:0), PG(P-20:0/0:0), PGE2, Reduced riboflavin, Riboflavin (Vitamin B2), Succinic anhydride, Xanthurenic acid were down-regulated. In serum (Fig. S13B), N-Acetyl-L-phenylalanine was down-regulated in BDE-3 groups. However, most metabolites were up-regulated including L-Proline, L-Valine, Ketoleucine, L-Leucine, 2-Phenylacetamide, L-Methionine, 4-Imidazolone-5-propionic acid, L-Carnitine, L-Phenylalanine, L-Tyrosine fragment, Phenyl sulfate, L-Tyrosine, 2-hendecenoic acid, L-Tryptophan fragment, Pyrocatechol sulfate, L-Tryptophan, Indoxylsulfuric acid, Dodecanedioic acid, Inosine, LysoPE(0:0/16:0), LysoPE(18:1/0:0), LysoPE(18:2/0:0), LysoPC(16:1), LysoPC(18:3), LysoPC(18:1), LysoPC(20:5/0:0), LysoPC(20:4), LysoPC(20:3), LysoPC(22:5) and Cholic acid glucuronide.

**Figure 6:**
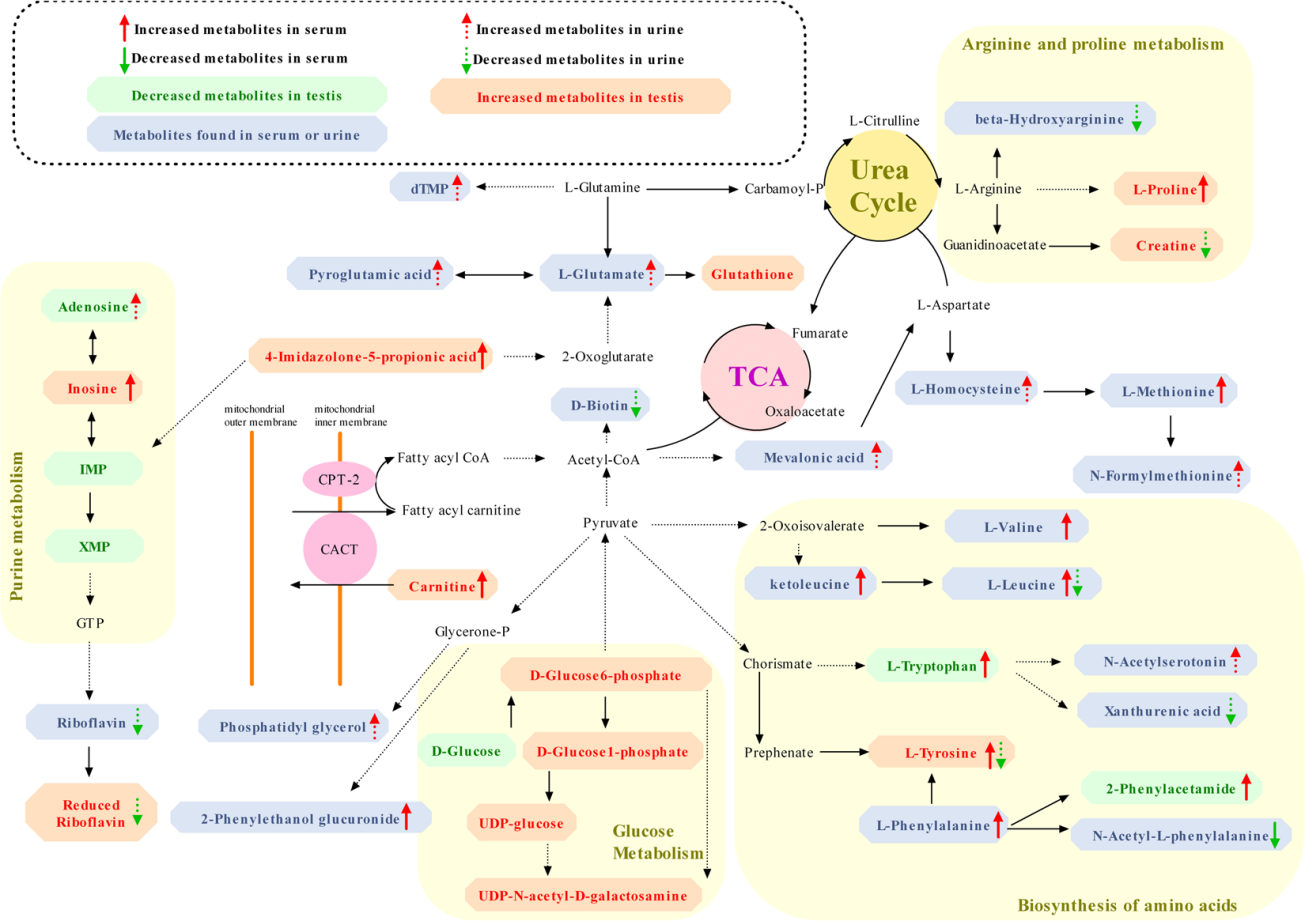
Metabolic pathways related to the differential metabolites identified in the BDE-3 groups.

The method applied to identify the differential metabolites was described above. Using this method, we identified a total of 76 differential metabolites in testis, 38 differential metabolites in urine and 31 differential metabolites in serum. The detailed information is shown in Table 1. Using the KEGG pathway database (http://www.genome.jp/kegg/)^23–25^, we constructed the metabolic network (Fig. 6) that clearly showed that BDE-3-induced toxicity was related to the alterations in the Arginine and proline metabolism, Valine, leucine and isoleucine degradation, Phenylalanine metabolism, Cysteine and methionine metabolism, Tyrosine metabolism, Purine metabolism, Pentose and glucuronate interconversions, Tryptophan metabolism, Biotin metabolism, Oxidative phosphorylation, Riboflavin metabolism, and Glycerophospholipid metabolism.

## Discussion

BDE-3 is the most fundamental mono-BDE which could be degraded from PBDEs in environment, although its bioaccumulation properties and potential healthy risk have raised concern, the effect on the adult male reproductive system are currently unclear. To our knowledge, it is the first report on assessment of male reproductive toxicity induced by mono-BDE in rodent. In this study, we found that short-term exposure to low-dose BDE-3 may have adverse effects on spermatogenesis in adult male mice. The sperm count decreased after the treatment of BDE-3 for 6 weeks at a very low dose (0.0015 mg/kg/day, a dose based on environmental exposure), and at the dose of ≥1.5 mg/kg/day, the sperm count decreased with statistical significance, and microscopical changes of germ cell loss in seminiferous tubules and epididymides were clearly observed at the dose of 30 mg/kg. Our previous studies on *C*. *Elegans* revealed that BDE-3 could induce ROS and DNA damage in germ cell^16^, which might be involved in the male reproductive toxicity that observed in mice.

Metabolomics has been applied in toxicity studies of chemicals as a newly emerging technology, especially in risk assessment of low dose exposure of environmental pollutants^26–29^, it may lead to critical insights because of its high sensitivity. Although several studies on toxicological effects of PBDEs using metabolomics analysis have been reported^30–32^, studies in rodent are limited and few data of mono-BDE is available. In this study, to obtain a better understanding of the toxic mechanism of BDE-3, we applied the UPLC-Q-TOF/MS profile to the metabolites of testicular tissue, urine and serum in the control and BDE-3 treated mice at different dosages.

In our study, we analyzed the testis tissue, serum and urine of BDE-3 treated mice. The differential metabolites from biological samples at different levels were screened and analyzed. As mentioned above, testis is susceptible to BDE-3 induced toxicity, so the metabolic alterations of testis tissue could, to some extent, reflect BDE-3 induced reproductive toxicity. Based on the pathway enrichment analysis on differential metabolites screened in testis tissue, we could preliminarily reveal that BDE-3 induced toxicity disturbed organism metabolic regulation network. Meanwhile, the metabolic alterations would spread through blood circulation and urine directly or indirectly. So pathway enrichment analysis and correlation analysis on differential metabolites screened in serum and urine could further clarify and verify the BDE-3 induced metabolic alterations. Besides, body fluid sample like serum and urine could reflect the systematic metabolic state of the body, so the differential metabolites which were highly correlated with BDE-3 induced toxicity screened in body fluid were expected to be potential toxic biomarker, improving the efficiency to detect the toxicity of PBDEs. In total, 76 differential metabolites in testicular tissue, 38 differential metabolites in urine and 31 differential metabolites in serum were identified. According to the score plots from the PLS-DA model of tissue and urinary samples, we can observed that the different BDE-3 groups showed a consistent separation trend from the control group. While as for serum samples, it had a minor pullback in the last BDE-3 group (30 mg/kg/day), and some metabolites corresponded with this variation trend, such as Inosine, L-Phenylalanine and some lipids metabolites (Fig. S14). We thought the possible reason is that the mice had developed resistance to the BDE-3 induced toxicity and self-healing capacity. Furthermore, the metabolic changes in urine was secondary to the changes in serum and target organs injury of BDE-3. That may be why the urine samples did not show a similar pullback change in the highest dose group.

### Nucleotides metabolism

Nucleotides play a key role as precursors of DNA and RNA, as activated intermediates in many biosynthetic processes, and as metabolic regulators. In the testis, nucleosides and nucleobases are important substrates of the salvage pathway for nucleotide biosynthesis^33^. We observed several purine and pyrimidine nucleobases metabolites in this study, such as Adenosine, Xanthine, Hypoxanthine and UDP-glucose, which significantly decreased in testicular tissue of BDE-treated mice compared with the control ones. Indicating that there will not be enough substrates for DNA and RNA biosynthesis.

In the mammalian spermatogenic pathway, Sertoli cells can provide nutrients and metabolic precursors to spermatogenic cells located within the blood-testis barrier (BTB)^34^. Because large amounts of nucleotides are required for spermatogenesis, less substrates will decelerate spermatogenesis. Accordingly, BDE-3 might affect the transportation of nucleobases and nucleosides across the BTB, or disturb the uptake of substrates by Sertoli cells. On the other hand, we observed several purine nucleobases metabolites, such as Adenosine and Inosine, significantly increased in serum and urine of BDE-treated mice compared with the control ones, which was potentially related to the reduction of purine and pyrimidine nucleobases metabolites level in the testis. Adenosine is an important endogenous nucleoside. Extracellular adenosine has the potential to influence the target cell metabolism in many aspects^35^. Adenosine can be phosphorylated to AMP, which can further generate Inosine by adenosine deaminase and IMP by AMP deaminase. The previous study had determined that Adenosine is also an important signaling molecule which is released under inflammatory conditions and there are four distinct subtypes are known, termed A1, A2A, A2B and A3^36^. It usually shows its endocrine effects including insulin and glucagon secretion interference by A1 receptor in mice^37^. Besides, Hiroyasu Nakata presented the first purification of a peripheral A1 adenosine receptor of rat testis membranes^38^. In this study, we observed reduction of nucleobases and nucleosides level in testis and the accumulation in serum and urine accordingly. Less nucleobases and nucleosides in testis will affect spermatogenesis and the extra purine nucleobases metabolites in serum will disturb the internal environment homeostasis, which was consistent with previous studies, indicating that the BDE-3 possibly disturbed the above purine and pyrimidine metabolism, and induced reproductive toxicity.

### Lipids metabolism

Lipids are abundant in testicles, which play an important role in membrane structure and function, energy storage and cell signaling^39^. Linoleic acid (LA) (18:2n-6) is the major Polyunsaturated fatty acid (PUFA) in vegetable oils and is a metabolic precursor to arachidonic acid (AA) (20:4n-6)^40^. A Previous report has indicated that LA family PUFA plays a criticle role in testicular function^41^. AA (20:4n-6) is a compound of important potent bioactivity, which mainly exists in phospholipid and LA mainly exists in phospholipid and triglyceride^41^. AA affects testis as a membrane component and providing energy storage, and a signal molecule in regulating steroidogenesis in Leydig cells as well^42^. In this study, decreased levels of LA and AA were found in BDE-3 exposed groups. Similarly, the increasing consumption of AA and LA level and corresponding ascending oxidative stress were found in testis after Bisphenol A exposure^43^. PUFA composition change observed in this metabolomics study was consistent with decreased spermatogenesis in this mice model. Therefore, testicular AA and LA alterations might be involved in BDE-3 testicular toxicity. Meanwhile, PUFA is very susceptible to peroxidation^44^. Thus, we speculated that the PUFA composition alterations indicated testicular oxidative stress.

We found significant increased GSH and L-carnitine levels in BDE-3 exposed group. The efficacy of l-carnitine and GSH as antioxidant substance has been confirmed both in humans and mice^45^. This kind of anti-oxidative composition might biologically adapt to oxidative stress^46^, thus the alterations was consistent with the hypothesis that testicular oxidative stress was increased in the BDE-3 exposed group. Oxidative stress could lead to both membrane lipid peroxidation and DNA fragmentation in testes^47^. It is reported that spermatogenesis^48^ and Leydig cell steroidogenisis^49^ are both vulnerable to oxidative stress^43^. There are clinical studies demonstrating that male infertility patients showed higher oxidative stress^50^. Additionally, oxidative stress and disturbed equilibrium of oxidant/antioxidant has been considered as a major mechanism of reproductive toxicity^51^. In present study, a possible explanation of decreased spermatogenesis of the mice model is oxidant/antioxidant imbalance, which has been well supported by population and animal studies^52,53^. Besides, in our study, a series of metabolites related to lipids metabolism such as lysophosphatidylcholine (LPC) and lysophosphatidyl ethanolamine (LysoPE) were detected, especially LPE significantly decreased in testicular tissue of the BDE-treated mice compared with the control group. LPE is an amphiphilic metabolite that is produced from membrane-phospholipids via the activation of phospholipase A2 (PLA2)^54^, and it also can synthesize PE under the action of LPCAT3 enzyme^55^. On the one hand, LysoPEs can stimulate invariant natural killer T cell activation through self-antigenicity, suggesting a possible role in innate immunity^56^, so we hypothesized that decreased LysoPEs in testicular tissue may cause its own immune disorder. On the other hand, it is essential that lysophospholipases (LysPLAs) and other phospholipases regulate phospholipid levels to maintain membrane homeostasis, flexibility and permeability^57,58^. This is critical to cell maintenance. In our study, the increase of various lipids metabolites potentially contributed to the BDE-induced toxicity.

### Other metabolic pathway

Riboflavin (vitamin B-2) belongs to micronutrients that play important roles in carbohydrate energy metabolism^59^. There are studies that suggest that the supplement of Ribovlavin, can significantly reduce the embryolethal action of methophenazine^60^. On the contrary, other studies suggest a syndrome, which included various limb, brain, orofacial, gastrointestinal, and miscellaneous malformations, can be induced by an intense riboflavin deficiency^61^. Besides, cytoflavin is proven to reduce the manifestations of mitochondrial dysfunction and the cell damage degree because of the antioxidant and membrane-protective properties^62^. In this study, the reduction of riboflavin in the BDE-3 groups, played an important role in the BDE-3 induced reproductive toxicity.

Several amino acids and their metabolites significantly changed in both blood and urine of the BDE-treated mice. They are mainly involved in arginine and proline metabolism, valine, leucine and isoleucine degradation, cysteine and methionine metabolism, phenylalanine metabolism, tyrosine metabolism, tryptophan metabolism, glycine, serine and threonine metabolism, D-Glutamine and D-glutamate metabolism, cysteine and methionine metabolism, especially valine, leucine and isoleucine biosynthesis. Arginine, one of the most metabolically versatile amino acids, is a semiessential or conditionally essential amino acid in humans and serves as a precursor for the synthesis of urea, nitric oxide, polyamines, proline, glutamate, creatine, and agmatine. Arginine is metabolized through a complex and highly regulated set of pathways^63^. Besides, the present studies found that the arginine-rich dipeptides, in particular Proline-Arginine (PR), are potently neurotoxic. The Proline-Arginine dipeptide (PR), is potently neurotoxic when expressed *in vivo* and neurons with nuclear PR aggregates have a much higher risk to undergo degeneration^64^. In this study, L-Proline is increased in serum of BDE-treated mice. Beta-Hydroxyarginine and creatine were decreased in urine. The BDE-induced toxicity was possibly related to the disturbed arginine and proline metabolism.

In addition, we observed that L-Tyrosine, L-Leucine and L-Isoleucine, significantly decreased in the urine, while they increased in the blood, in the BDE-treated mice. These results suggested that the thiamine metabolism, valine, leucine and isoleucine degradation, and especially the polarity and clearance of the above metabolites are disturbed during the formation of HLP-induced toxicity, thus resulting in the abnormalities of body’s reproductive system metabolism.

## Materials and Methods

### Chemicals and reagents

Methanol (HPLC grade), acetonitrile (HPLC grade), formic acid (HPLC grade), and 2-chloro-L-phenylalanine (as an internal standard) were purchased from Sigma. (Saint Louis, USA). Ultrapure water was produced using the Milli-Q system (Millipore, Bedford, MA, USA). Hematoxylin-eosin staining kit was purchased from Shanghai Ailex Technology Co., Ltd.BDE-3 (CAS 101-55-3, 99% purity) was purchased from Sigma-Aldrich (St. Louis, MO).

### Animals and treatment

The C57BL/6J gpt delta mice were kindly provided by Dr. Nohmi of the National Institutes of Health Sciences, Japan. Animals were acclimatized in Specific Pathogen Free rooms with the temperature at 20–26 °C, the humidity at 30–70% and a 12-h light/dark cycle for at least 1 week. Regular laboratory chow and filtered tap water were allowed adlibitum. All animal experiments were approved by the National Institutes of Health Guide for the Care and Use of Laboratory Animals with the approval (SYXK‐2013‐0050) of the Scientific Investigation Board of Shanghai Jiao Tong University School of Medicine, Shanghai, China. All animal experiments were approved (A-2016–004) by Department of Lab Animal Science and The Animal Care and Welfare Committee of Shanghai Jiao Tong University School of Medicine, Shanghai, China. All experiments were performed in accordance with Standard operation procedure and relevant guidelines and regulations of Shanghai Jiao Tong University School of Medicine, Shanghai, China.

In this study, we examined the effects of BDE-3 on the reproductive function in mice, with the lowest dose of 0.0015 mg/kg/day, which equal to the highest ingestion concentration of PBDEs in human (141 ng/kg/day) by the U.S. Environmental Protection Agency (EPA), 1.5 mg/kg/day, an equal dose to the lowest effective dose of its congener of BDE-471, middle dose of 10 mg/kg/day and high dose of 30 mg/kg/day, respectively.

Fifteen-weeks-old male mice were randomly allocated to 5 groups (n = 6 per group) and treated intragastrically with BDE-3 dissolved in corn oil 6 days/week for consecutive six weeks at different dosages of 0.0015, 1.5, 10 and 30 mg/kg. The vehicle control group received corn oil at the same manner. Mice were forced into metabolismcage for 24 hours once every two weeks to facilitate the collection of urine, the collections were immediately centrifuged at 4000 rpm for 5 min, and the supernatant was stored at −80 °C until being analyzed. Twenty-four hours after the last treatment, the mice were anesthetized by chloral hydrate. The blood samples were collected via eyeball and clotted at 4 °C for 40–60 minutes and then centrifuged at 4000 rpm for 5 minutes to obtain serum. The serum samples were stored at −80 °C until analysis. After blood collection, the epididymis and testis were dissected and weighed (wet weight). We collected the urine at different time points, but to match our serum samples (collected at 6th week), we did metabolomics profiling analysis only using the urine which were collected at 6th week. The remaining urine samples might be used for some deeper researches in the future.

### Sperm count, vitality and morphology analysis

For sperm count analysis, one epididymis from each individual was put in a 1.5 mL tube with 37 °C pre-warmed M199 (containing 0.5% Fetal Bovine Serum) and cut into pieces for the preparation of sperm suspension. A drop of sperm suspension was put on a counting plate to measure the sperm count and sperm vitality. For sperm vitality examination, at least 200 sperm were checked and divided into 4 categories: I-fast straight forward movement, II-slow forward movement or rotation, III-*in situ* fibrillation or spinning, IV-no movement. Rate of sperm vitality % = (I + II + III)/(I + II + III + IV) × 100%. For morphological examination, 1–2 drops of sperm suspension were smeared on a slide. After drying, the slides were fixed in methanol for 5 minutes and stained with eosin for about 60 minutes before examined. At least 500 intact sperm per individual were examined under the microscope to calculate the ratio of abnormality. The abnormality was divided for 6 types: banana gate, enlarged-headed, amorphous, tail fold, double-headed and double-tailed. Rate of sperm abnormality (%) = total deformed sperm/total observed sperm × 100%.

### Histopathology

One testis from each animal was fixed in modified Davidson solution and one epididymis of each animal was fixed in 10% neutral formalin fixative. After being well fixed, the tissues were sampled, dehydrated in automatic tissue hydroextractor (Pathcentre, Thremo) and embedded in paraffin embedding machine (Shandon Histocentre 2, Thremo) and then cut into 3 µm paraffin section in microtome (Shandon Finesse E+, Thremo). The sections were stained with hematoxylin and eosin. All tissues were analysised by manual check and scanned by slice scanning system (Scan ScopeXT, Aperio).

### UHPLC-Q-TOF/MS metabolomics analysis

The frozen urine and serum samples were thawed at room temperature. 300 μL methanol solution that contained 5 μg/mL 2-Chloro-L-phenylalanine as the internal standard was added into 100 μL urine or serum samples. The mixed samples were homogenized by vortex for 5 min and then were placed at room temperature for 10 min and centrifuged at 13,000 rpm, 4 °C for 15 min. 150 μL of supernatant were added into the sample vials.

The frozen testicular tissue samples were thawed at room temperature. Then they were weighed, and homogenized in 900 μl 80% methanol solution that contained 5 μg/mL 2-chloro-l-phenylalanine as an internal standard. Then the mixture were centrifuged at 13,000 rpm, 4 ◦C for 15 min. 150 μl of supernatant were added into the sample vials.

A quality control sample (QC) was prepared by mixing the same volume from all supernatant samples (10 μL from each sample). The QC sample was run six times prior to the start of the analytical run to “condition” the system and analyzed after every 9 samples to check the system stability^65^.

UHPLC-Q-TOF/MS analysis was performed using an Agilent 1290 Infinity LC system coupled to an Agilent 6538 Accurate-Mass Quadrupole Time-of-Flight (Q-TOF) mass spectrometer (Agilent, USA). Chromatographic separations were performed at 25 °C using a Waters XSelect® HSS T3 analytical column (2.1 mm × 100 mm, 2.5 μm, Waters, Milford, MA). The flow rate was 0.4 mL/min and the injection volume was 3 μL. The mobile phase consisted of 0.1% formic acid (A) and ACN modified with 0.1% formic acid (B). The gradient elution conditions for urinary samples were 5% B at 0–1 min, 5–20% B at 1–6 min, 20–50% B at 6–9 min, 50–95% B at 9–13 min, 95% B at 13–15 min and followed by re-equilibrated step of 5 min. As for serum samples, the following gradient program was used: 5%B at 0–2 min, 5–95%B at 2–17 min, 95%B at 17–19 min. The gradient elution conditions for tissue samples were 5% B at 0–2 min, 5–95% B at 2–13 min, 95% B at 13–15 min. The capillary voltage was 4 kV in positive mode and 3.5 kV in negative mode, the drying gas flow was 11 L/min, and the gas temperature was 350 °C. The nebulizer pressure was set at 45 psig. The fragmentor voltage was set at 120 V and skimmer voltage was set at 60 V. The reference ions were 121.0508 and 922.0098 in positive mode, 119.03632 and 966.000725 in negative mode. Data were collected in a centroid mode and the mass range was set at m/z 50–1100 using an extended dynamic range. MS/MS analysis was carried out to study the structure of the potential biomarkers and the collision energy was range from 10 to 40 eV.

### Data processing and statistical analysis

In the analysis of body weight, testis and epididymis weight, sperm count, vitality and morphology, the Dunnett-t test was used to determine significant differences between BDE-3 exposure groups and the vehicle control group after the one-way ANOVA was performed. A value of P < 0.05 was considered significant. SPSS version 17.0 (IBM, USA) was used for statistical analysis.

The raw UHPLC–MS data were transformed into a common data file format (.mzdata) using MassHunter Qualitative software. The interferences of the isotopes were excluded and the absolute peak height was set at 300 counts. In the R software platform, the XCMS program was applied for peak extraction, peak alignment, and automatic integration^66^. The 80% rule was used to filter the ions and the remaining ions were then normalized to internal standard peak area. The Retention time (Rt)-m/z pairs, observations, and relative ion intensities were imported into SIMCA-P 11.0 software package (Umetrics, Umea, Sweden) for the multivariate statistical analysis. Prior to multivariate analysis, the resultant data matrices were mean-centered and scaled to Pareto variance. Unsupervised principal component analysis (PCA) and partial least squares discriminate analysis (PLS-DA) were applied to observe the stability of the assay in the sequence, the separating trends, and further identify the metabolite candidates contributing to the clustering of samples. We applied variable importance plot (VIP) representing the confirmation of the importance or power of the selected candidates to select metabolite candidates with the threshold value of 1. In addition, the model of PLS-DA was evaluated according to the cross-validation of R2, Q2 value and permutation test. An independent sample t-test was performed for the statistical analysis using SPSS version 17.0 (IBM, USA) and p < 0.05 was considered statistically significant.

### Pathway enrichment analysis

We applied the Pathway Analysis module on Metaboanalyst website (http://www.metaboanalyst.ca/). We firstly import the differential metabolites of testis tissue, serum, and urine to this website, matching the HMDB, PUBCHEM and KEGG database (The mass error tolerance was “<15ppm”), and the metabolites were categorized based on the link from KEGG. Select mouse as species to do pathway enrichment analysis. The results were shown on Fig. S15. Then manually construct the pathway network based on the KEGG pathways we have got, and some important pathways included were shown on Fig. S16.

## Conclusion

In this study, we successfully observed the reproductive toxicity induced by BDE-3 in mice via clinical observation, Sperm count and vitality, Sperm morphology and Histopathology examination. A new scientific metabolomic method based on UPLC-Q-TOF/MS was applied to profile the differential metabolites in testis, urine and serum in the control and BDE-3 treated mice at different dosages. In total, 76 differential metabolites in testicular tissue, 38 differential metabolites in urine and 31 differential metabolites in serum were identified. Pathway analysis revealed that several pathways were potentially related to BDE-3 induced toxicity including Arginine and proline metabolism, Valine, leucine and isoleucine degradation, Phenylalanine metabolism, Cysteine and methionine metabolism, Tyrosine metabolism, Purine metabolism, Pentose and glucuronate interconversions, Tryptophan metabolism, Biotin metabolism, Oxidative phosphorylation, Riboflavin metabolism, and Glycerophospholipid metabolism. These findings demonstrated the BDE-3 induced reproductive toxicity in mice. Our study may be helpful to arouse our awareness of the toxicity of PBDEs and to give a possible explanation for the mechanism, which indicates that UHPLC-Q-TOFMS-based metabolomics approach allowed a better understanding of PBDEs-induced toxicity dynamically.

### Data Availability

The datasets generated and analysed during the current study are available from the corresponding author on reasonable request.

## Electronic supplementary material

Supplementary Information
