# Supplementary material for: Metabolomics coupled with pathway analysis characterizes metabolic changes in response to BDE-3 induced reproductive toxicity in mice
Source: Sci Rep. 2018 Apr 3;8:5423. doi: 10.1038/s41598-018-23484-2 (PMC5882662; doi:10.1038/s41598-018-23484-2)
Supplement: Supplementary file 1 — Supplementary Information [file 41598_2018_23484_MOESM1_ESM.doc]

**Metabolomics coupled with pathway analysis characterizes metabolic changes in response to BDE-3 induced reproductive toxicity in mice**

**Ziheng Wei, ^1¶^ Jing Xi, ^2¶^ Songyan Gao, ^3¶^ Xinyue You, ^2^ Na Li, ^3^ Yiyi Cao, ^2^ Liupeng Wang, ^2^ Yang Luan ^2*^ and Xin Dong ^3*^**

^1^. Faculty of Naval Medicine, Second Military Medical University, Shanghai 200120, P. R. China. E-mail: wzh_smmu@163.com.

^2^. Hongqiao International Institute of Medicine, Shanghai Tong Ren Hospital and Faculty of Public Health, Shanghai Jiao Tong University School of Medicine, Shanghai, 200025, P. R. China

^3^. School of Pharmacy, Second Military Medical University, Shanghai 200120, P. R. China. E-mail: [sy_gaosmmu@163.com](mailto:sy_gaosmmu@163.com),

^*^. Corresponding authors

[dongxinsmmu@126.com](mailto:dongxinsmmu@126.com)(Xin Dong ) and [yluan@sjtu.edu.cn](mailto:yluan@sjtu.edu.cn) (Yang Luan)

**^¶^**. Ziheng Wei, Jing Xi and Songyan Gao contributed equally to this work.

**Figure S1.** Body weight of mice treated with different concentration of BDE-3, mean ± SD (n=6). No significant differences were observed.

**Figure S2.** Epididymis (A) and testis (B) coefficient of mice treated with different concentration of BDE-3, mean ± SD (n=6). No significant differences were observed.

**Figure S3.** Rate of banana gate sperm(A) , enlarged-headed sperm(B) , amorphous sperm (C), double-headed/tailed sperm(D) and headless sperm(E) at the mice treated with different concentration of BDE-3, mean ± SD (n=6). No significant differences were observed.

**
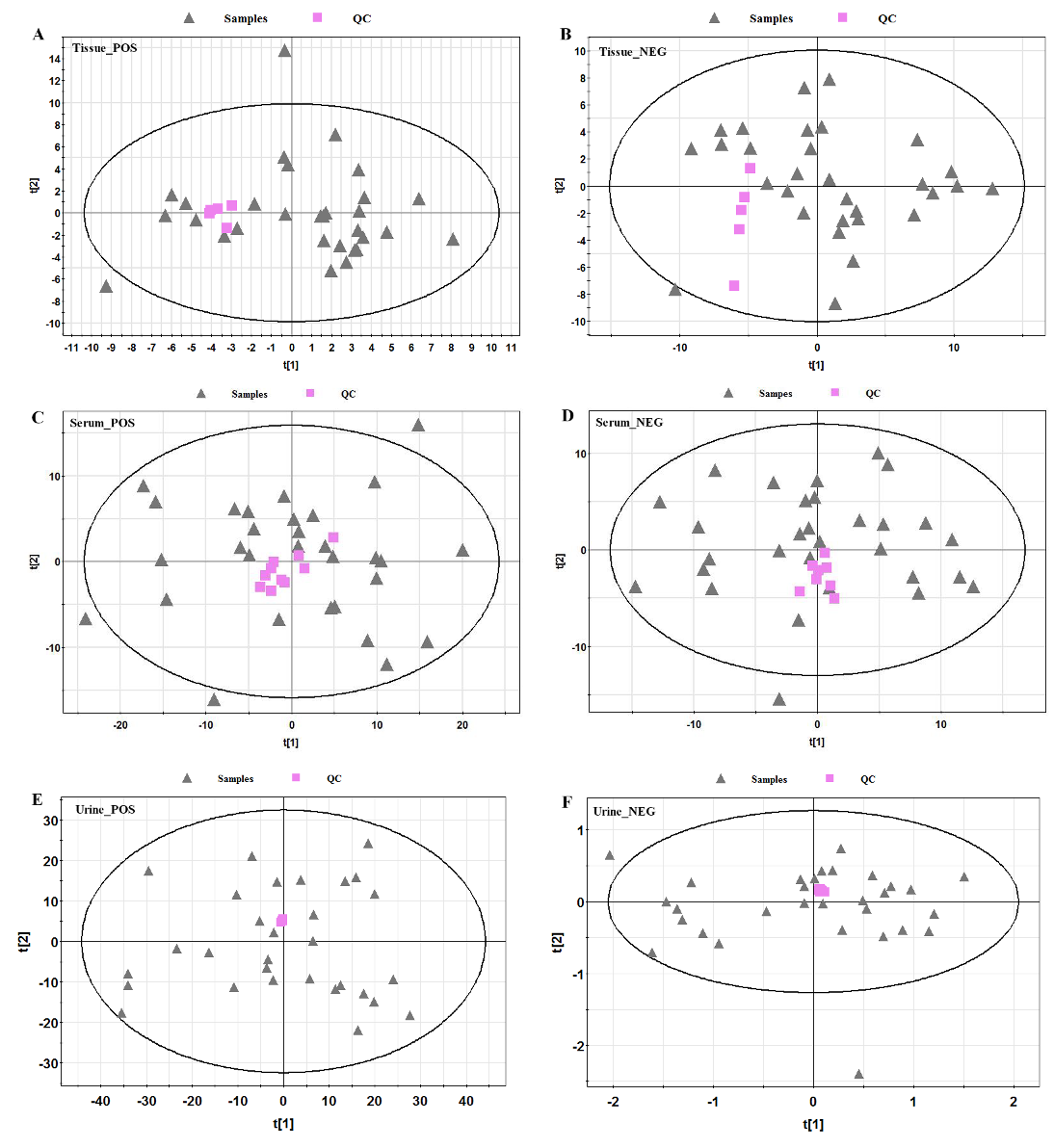
**

**Figure S4.** PCA score plots of quality control (QC) samples and other testicular, urinary or serum samples in different groups in positive and negative mode.

**
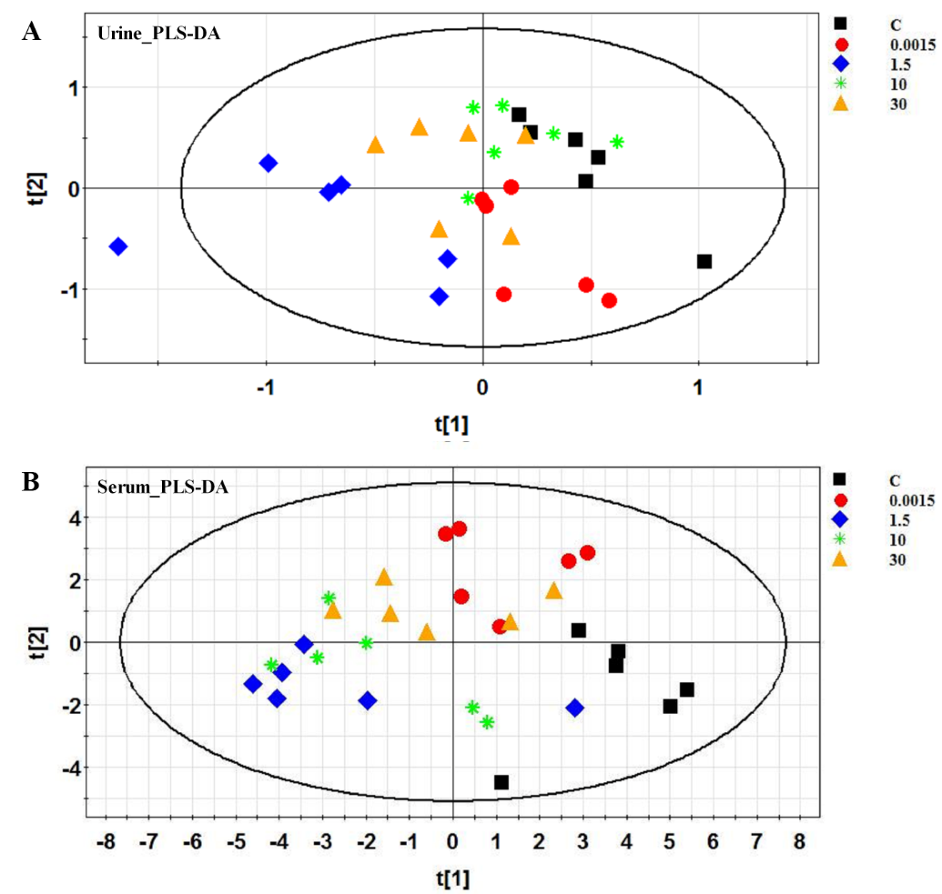
**

**Figure S5.** PLS-DA score plots of urine (A) and serum (B) samples in the control group and BDE-3 groups at different dosages by RPLC-MS methods in negative mode. (A) Urine samples of control group and all BDE-3 groups based on RP-MS; (B) Serum samples of control group and all BDE-3 groups based on RP-MS.


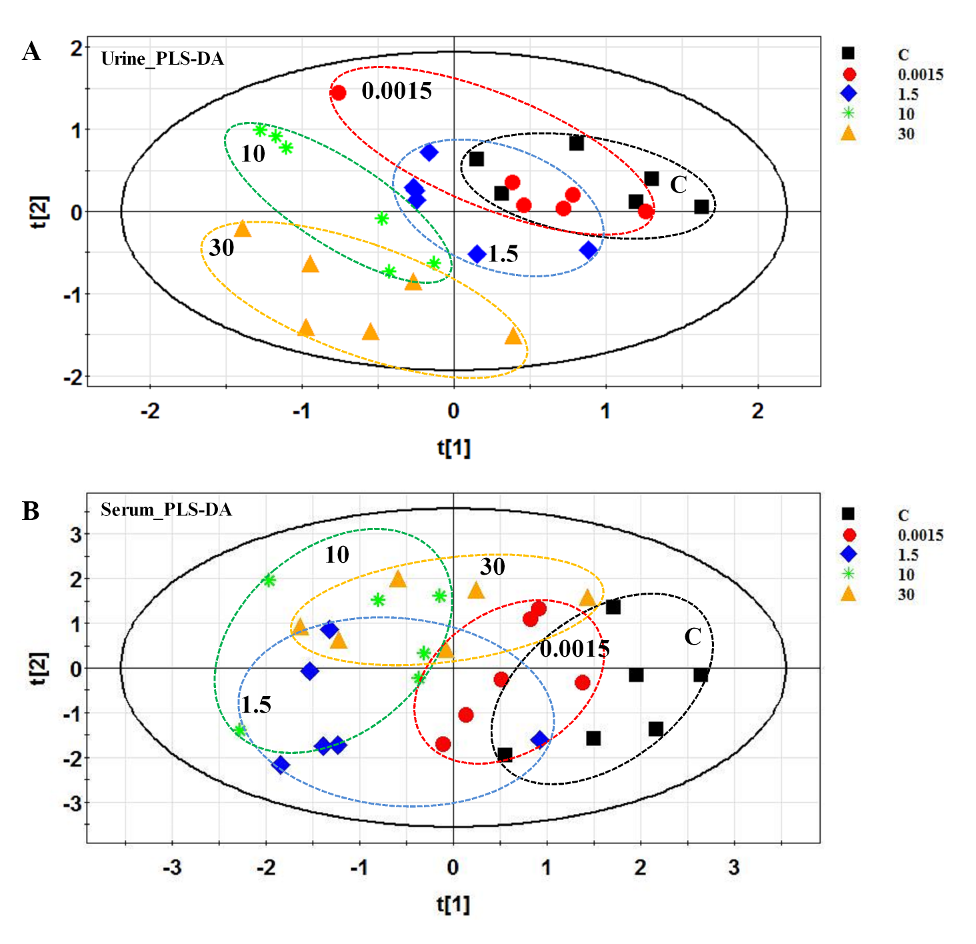


**Figure S6.** PLS-DA score plots of urine (A) and serum (B) samples in the control group and BDE-3 groups at different dosages by RPLC-MS methods in positive mode. (A) Urine samples of control group and all BDE-3 groups based on RP-MS; (B) Serum samples of control group and all BDE-3 groups based on RP-MS.


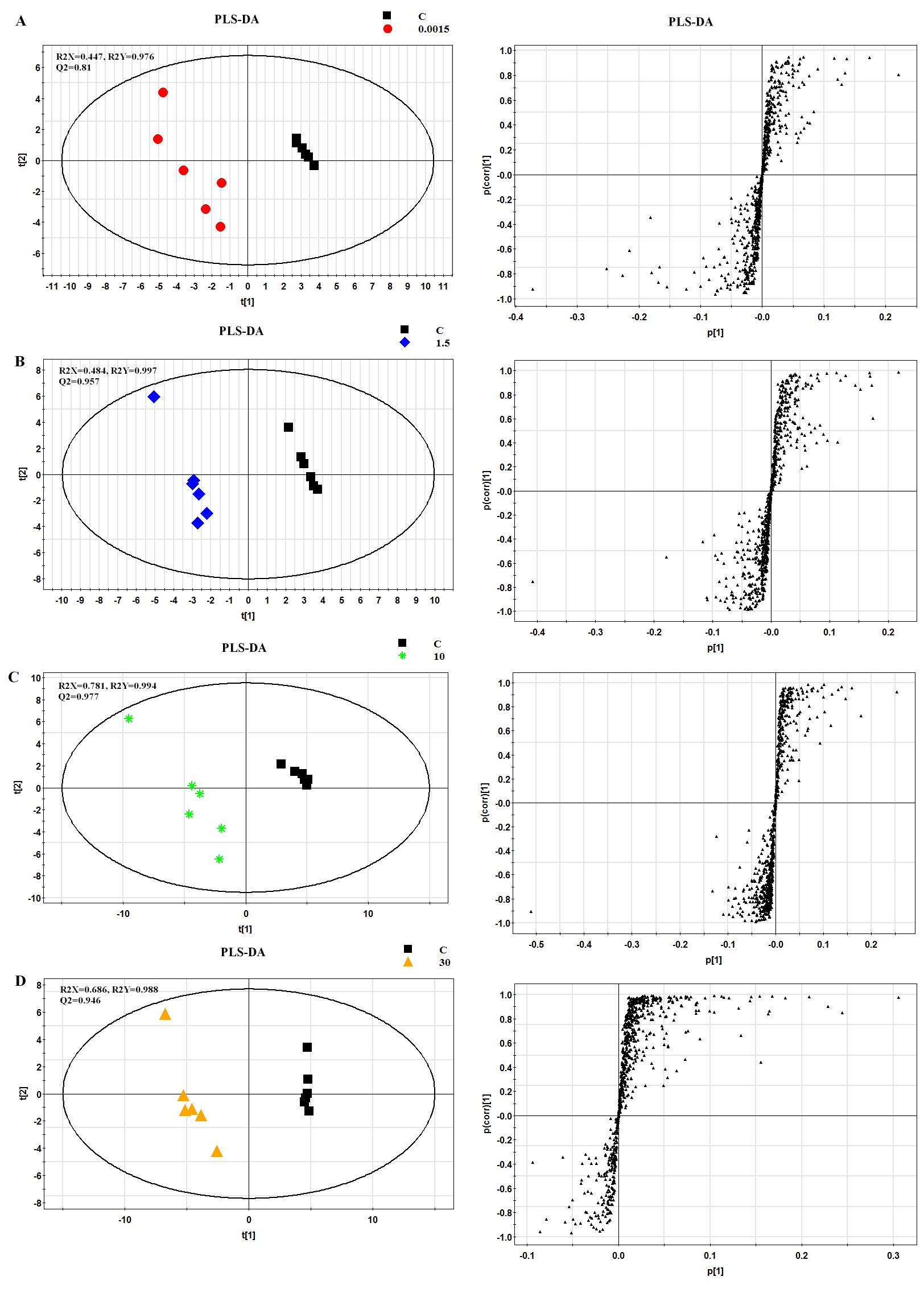


**Figure S7.** PLS –DA score plots of testis in the control and each different BDE-3 groups at different dosages (A-D) by RPLC-MS methods in positive mode and the corresponding S-plot (E-F).


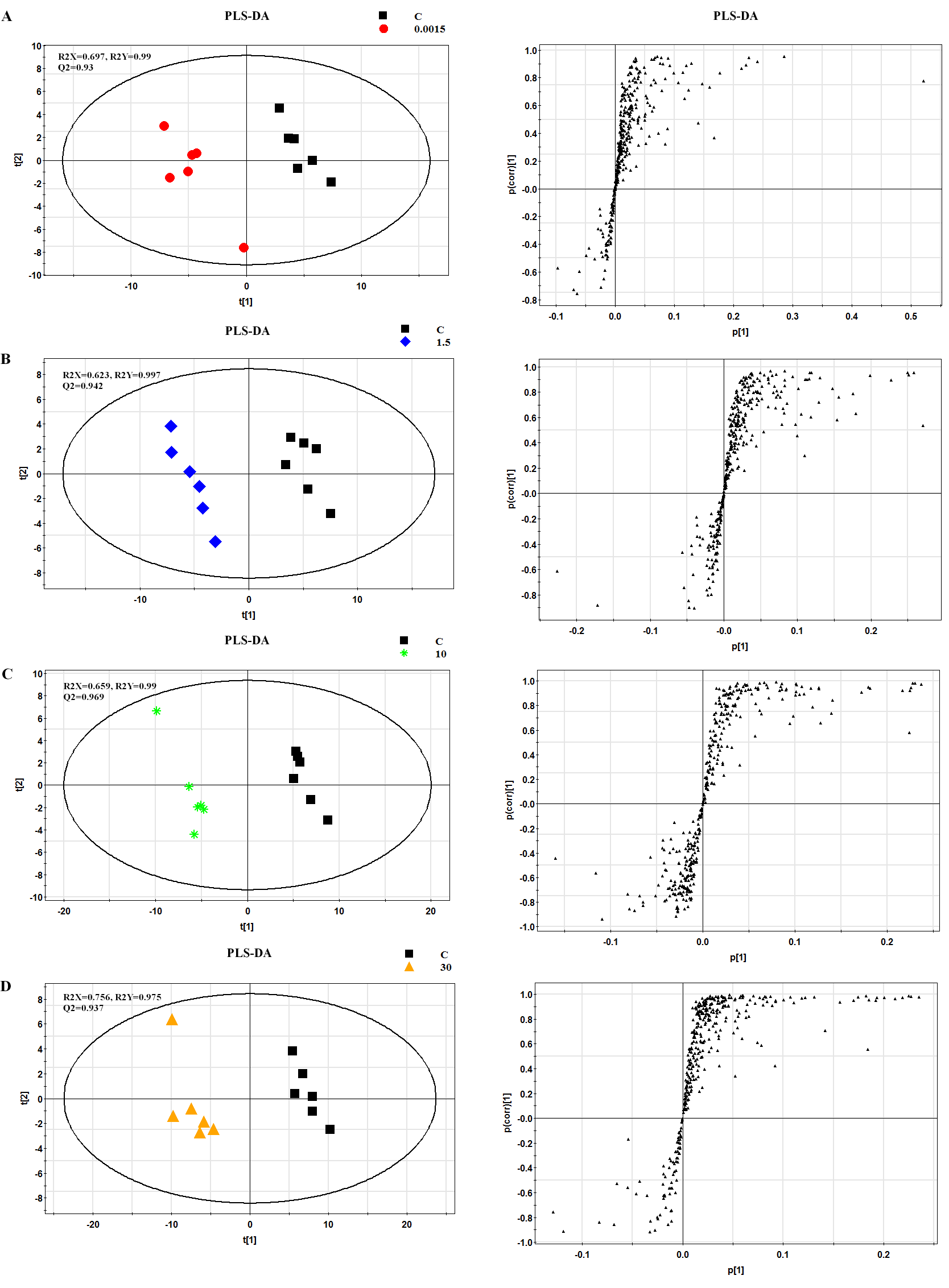


**Figure S8.** PLS –DA score plots of testis in the control and each different BDE-3 groups at different dosages (A-D) by RPLC-MS methods in negative mode and the corresponding S-plot.

**
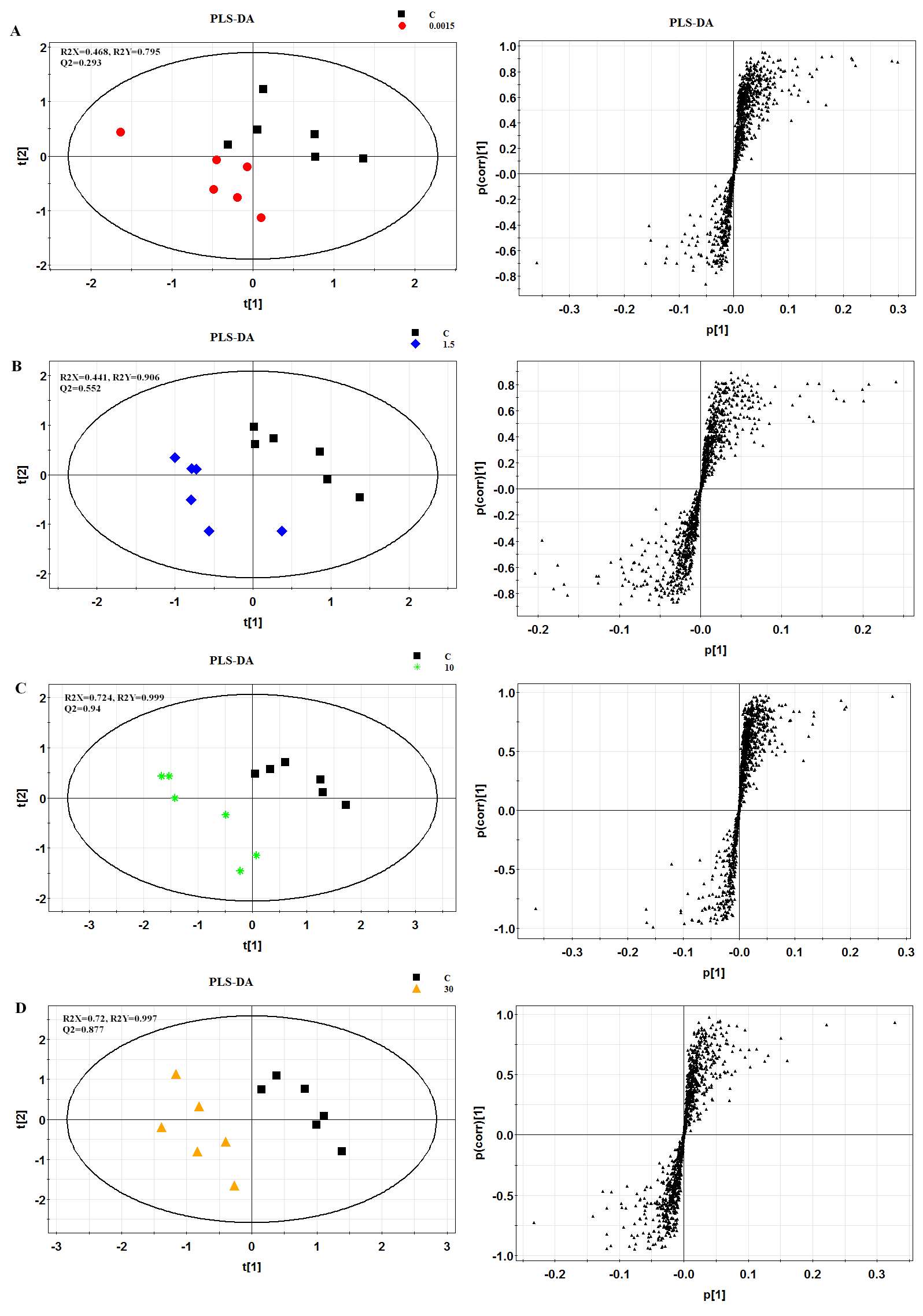
**

**Figure S9.** PLS –DA score plots of urine in the control and each different BDE-3 groups at different dosages (A-D) by RPLC-MS methods in positive mode and the corresponding S-plot.


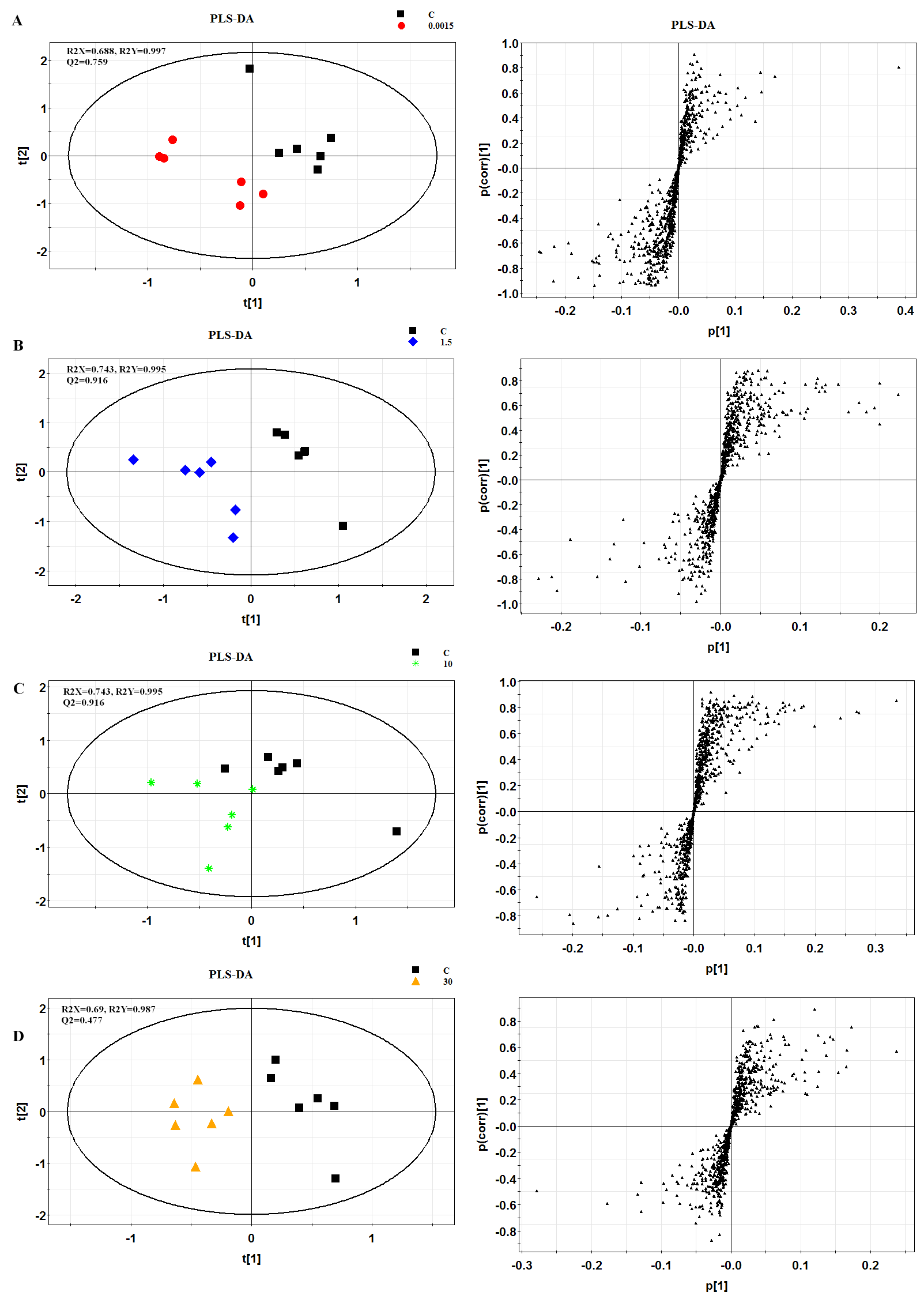


**Figure S10.** PLS –DA score plots of urine in the control and each different BDE-3 groups at different dosages (A-D) by RPLC-MS methods in negative mode and the corresponding S-plot.


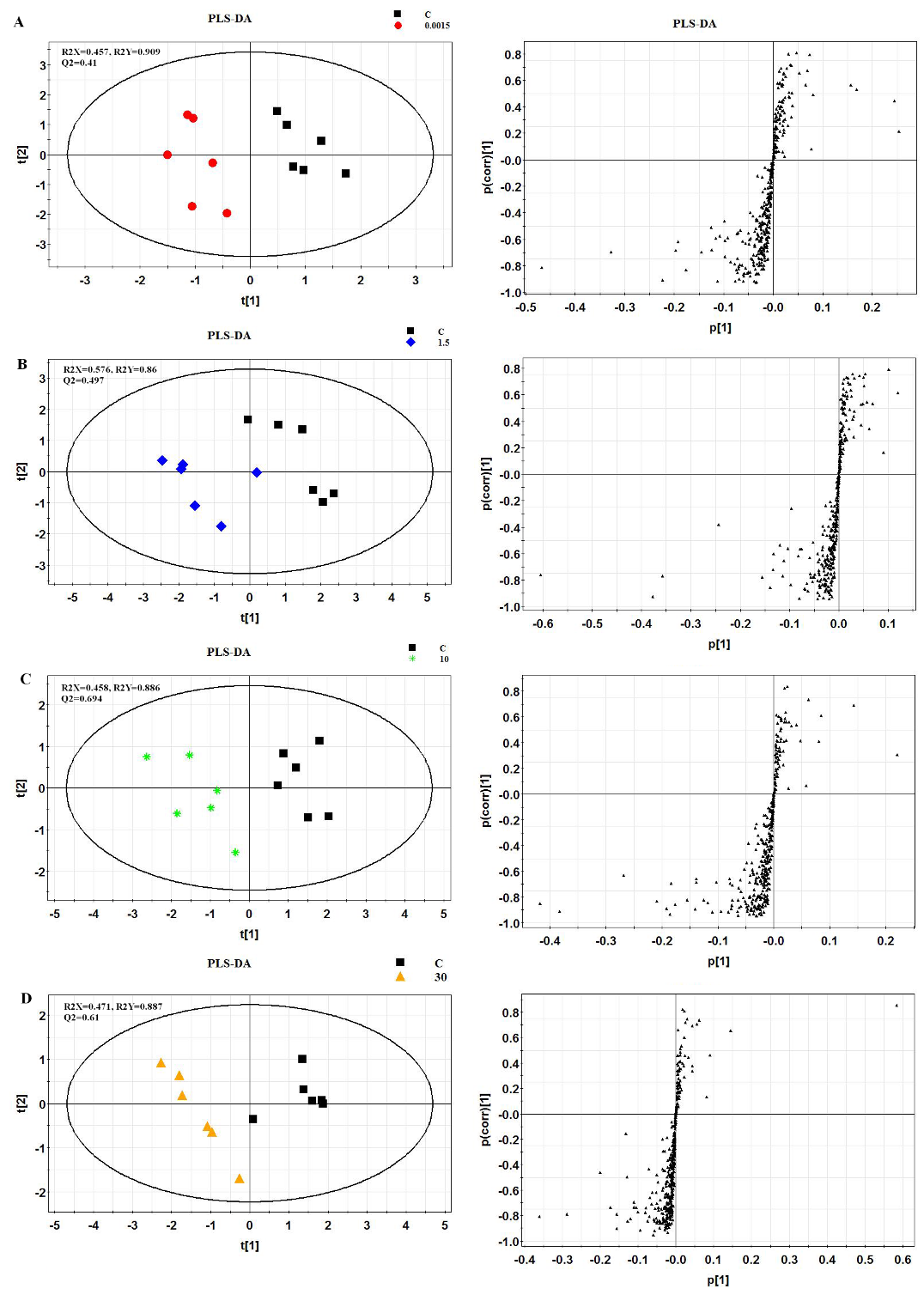


**Figure S11.** PLS –DA score plots of serum in the control and each different BDE-3 groups at different dosages (A-D) by RPLC-MS methods in positive mode and the corresponding S-plot.


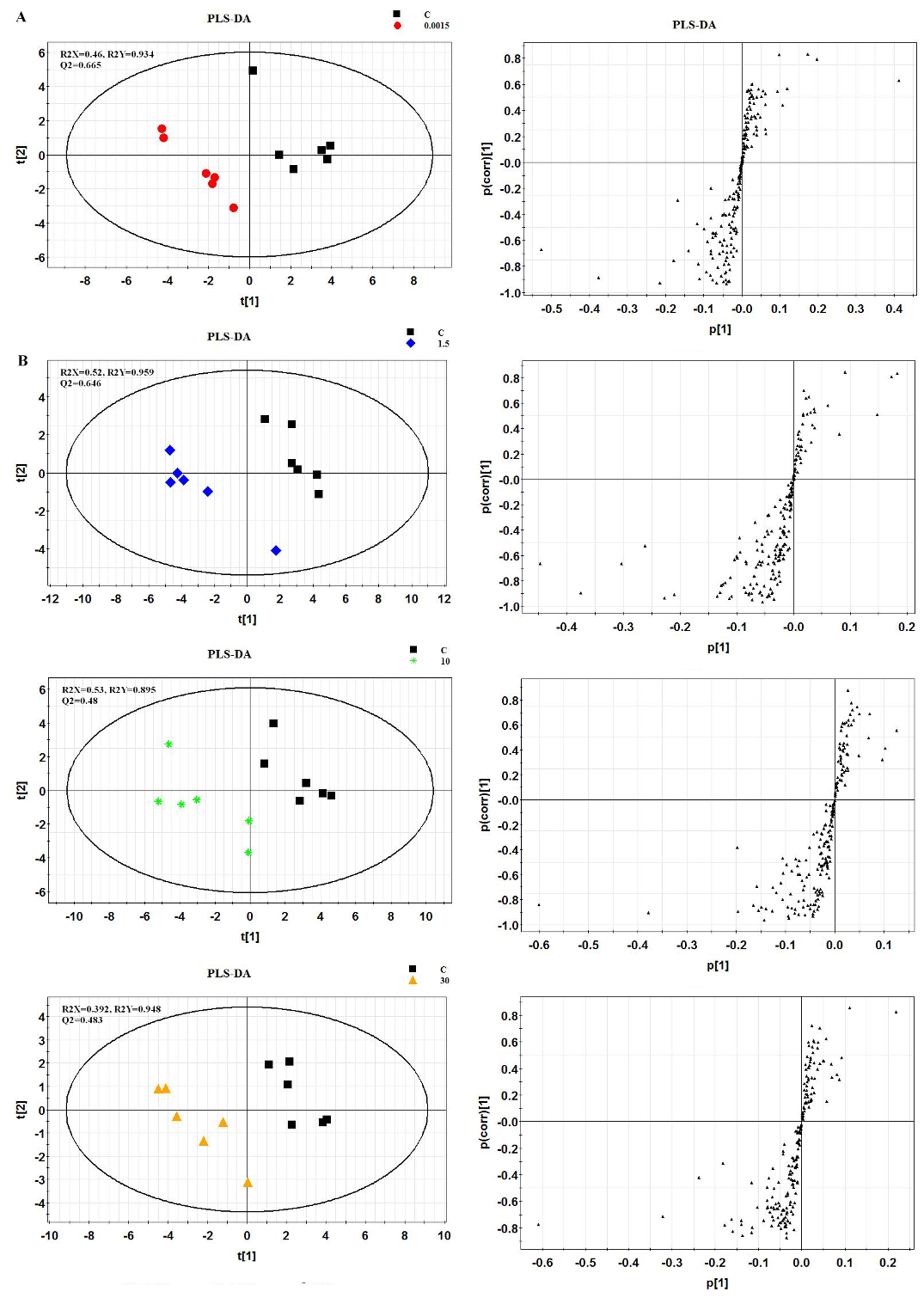


**Figure S12.** PLS –DA score plots of serum in the control and each different BDE-3 groups at different dosages (A-D) by RPLC-MS methods in negative mode and the corresponding S-plot.

**Figure S13. (A)**


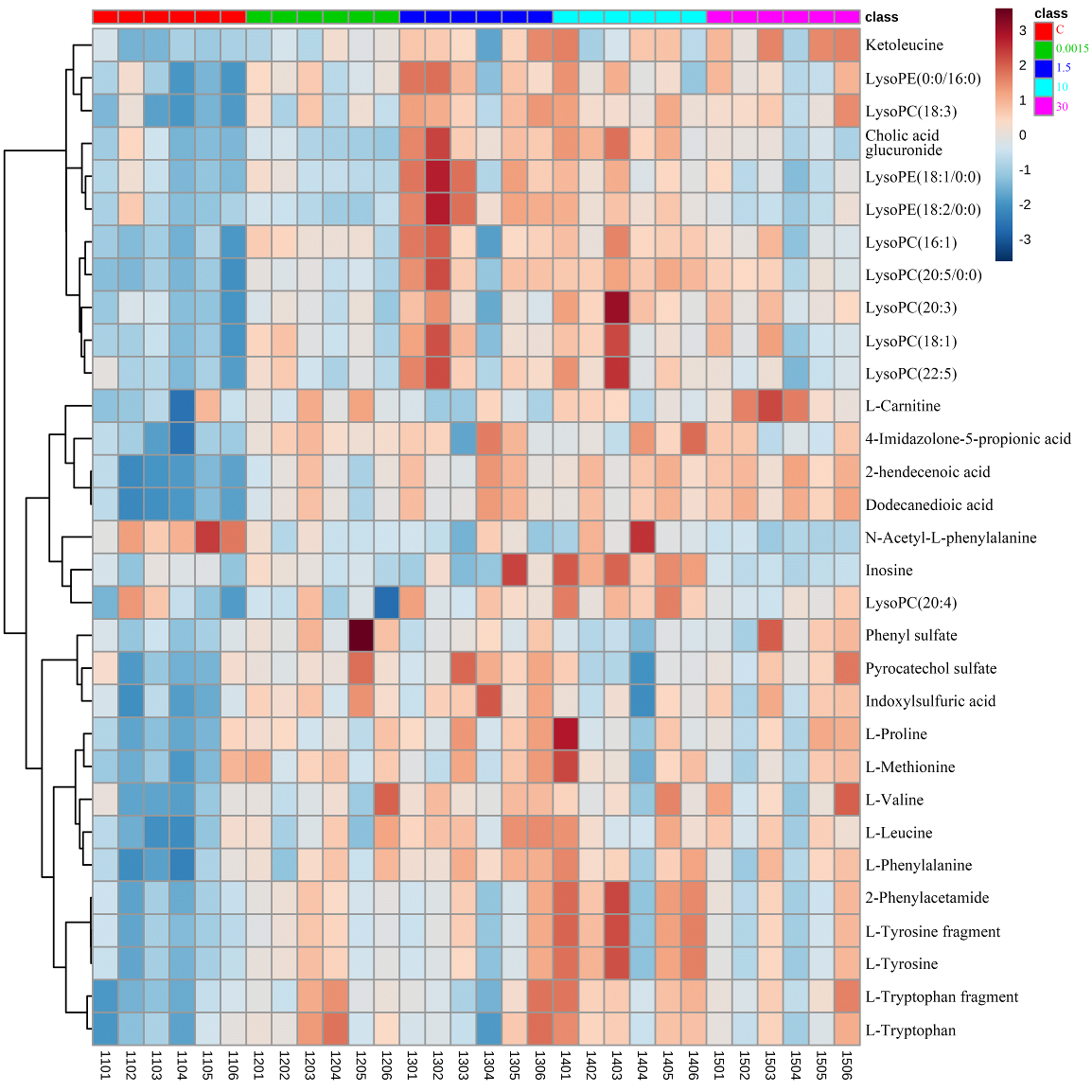


**Figure S13. (B)**


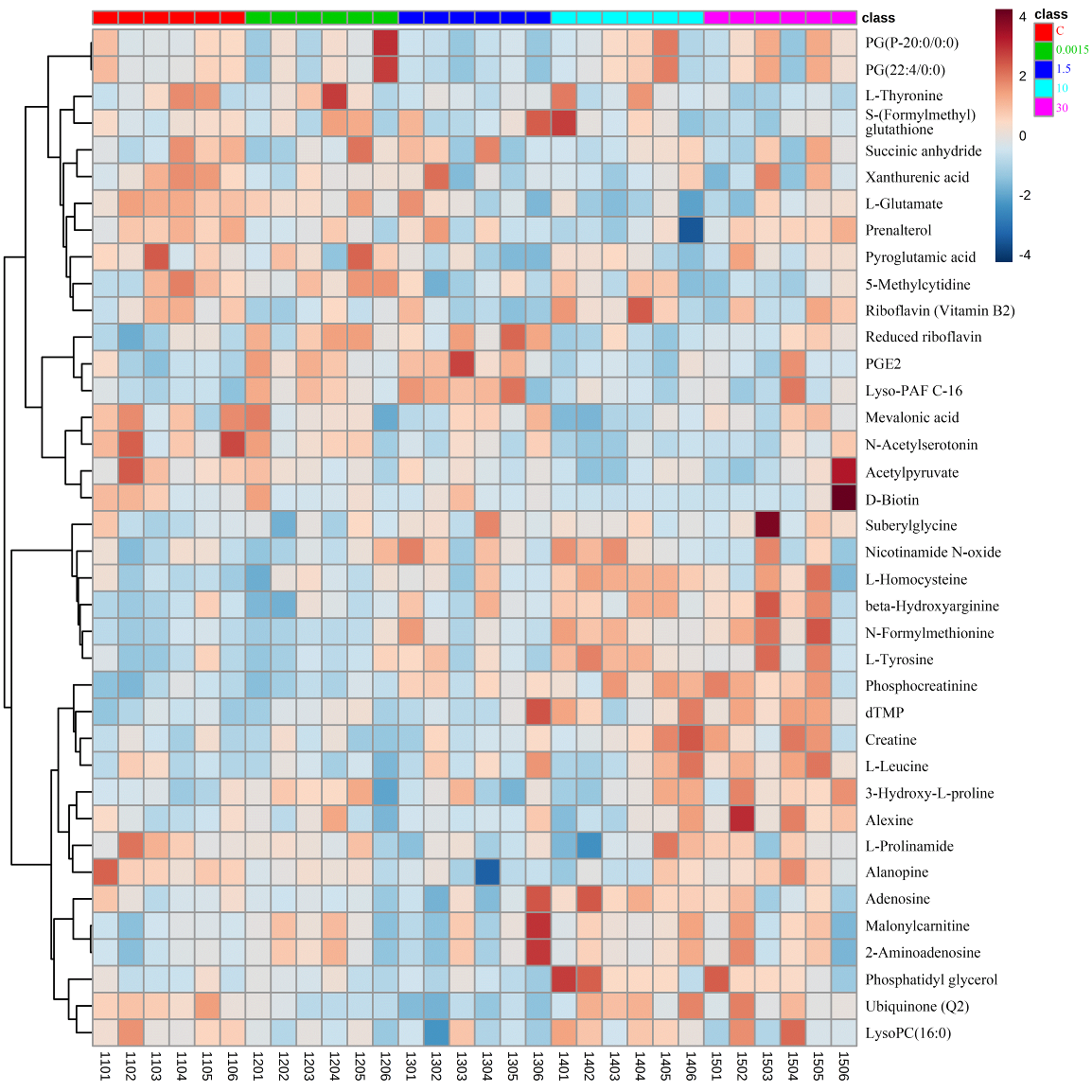


**Figure S13.** The clustering heat map of the control (C) and BDE-3 mice across different dosages (0.0015, 1.5, 10, 30) based on the 38 and 31 differentially metabolites in urine (A) and serum (B), respectively. Each column is labeled with different colors according to the sample type.


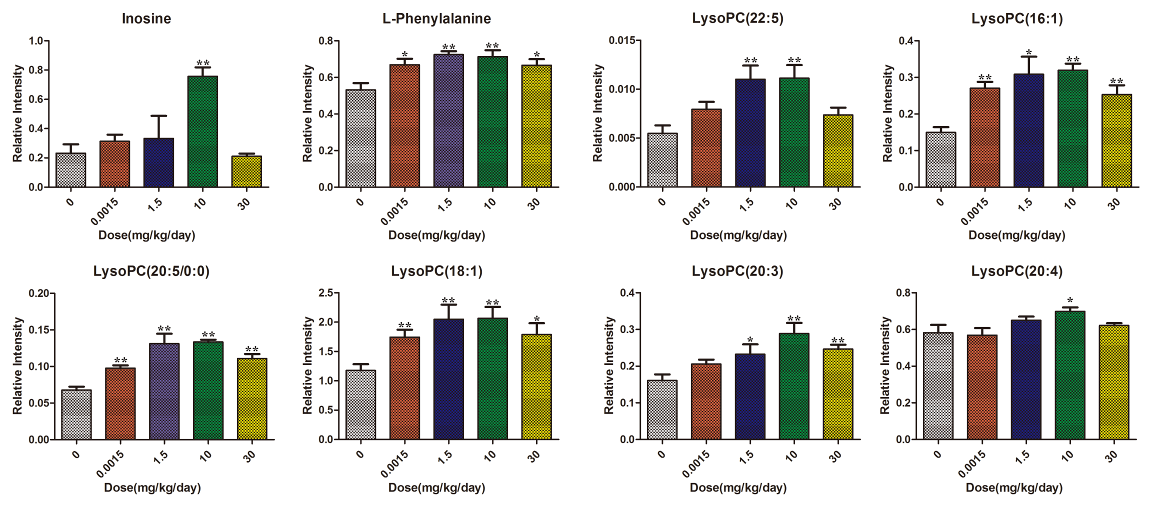


**Figure S14.** Variation trend of metabolites which had a minor pullback in the last BDE-3 group (30 mg/kg/day), mean ± SD (n=6). *p<0.05 versus solvent group, **p<0.01 versus solvent group.


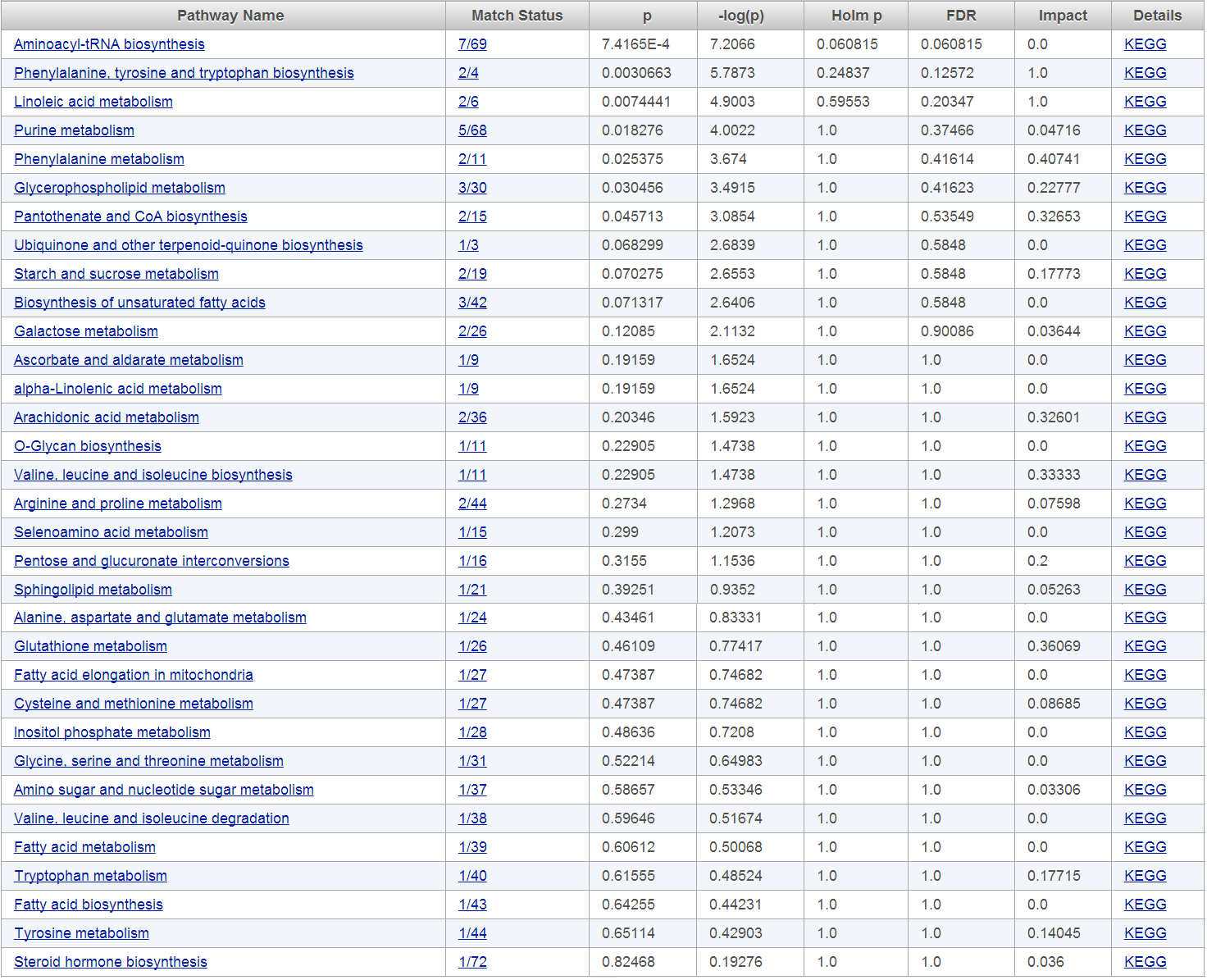


**Figure S15.** The pathway enrichment analysis results of metabolites in testis tissue, serum, and urine based on the Pathway Analysis module on Metaboanalyst website.


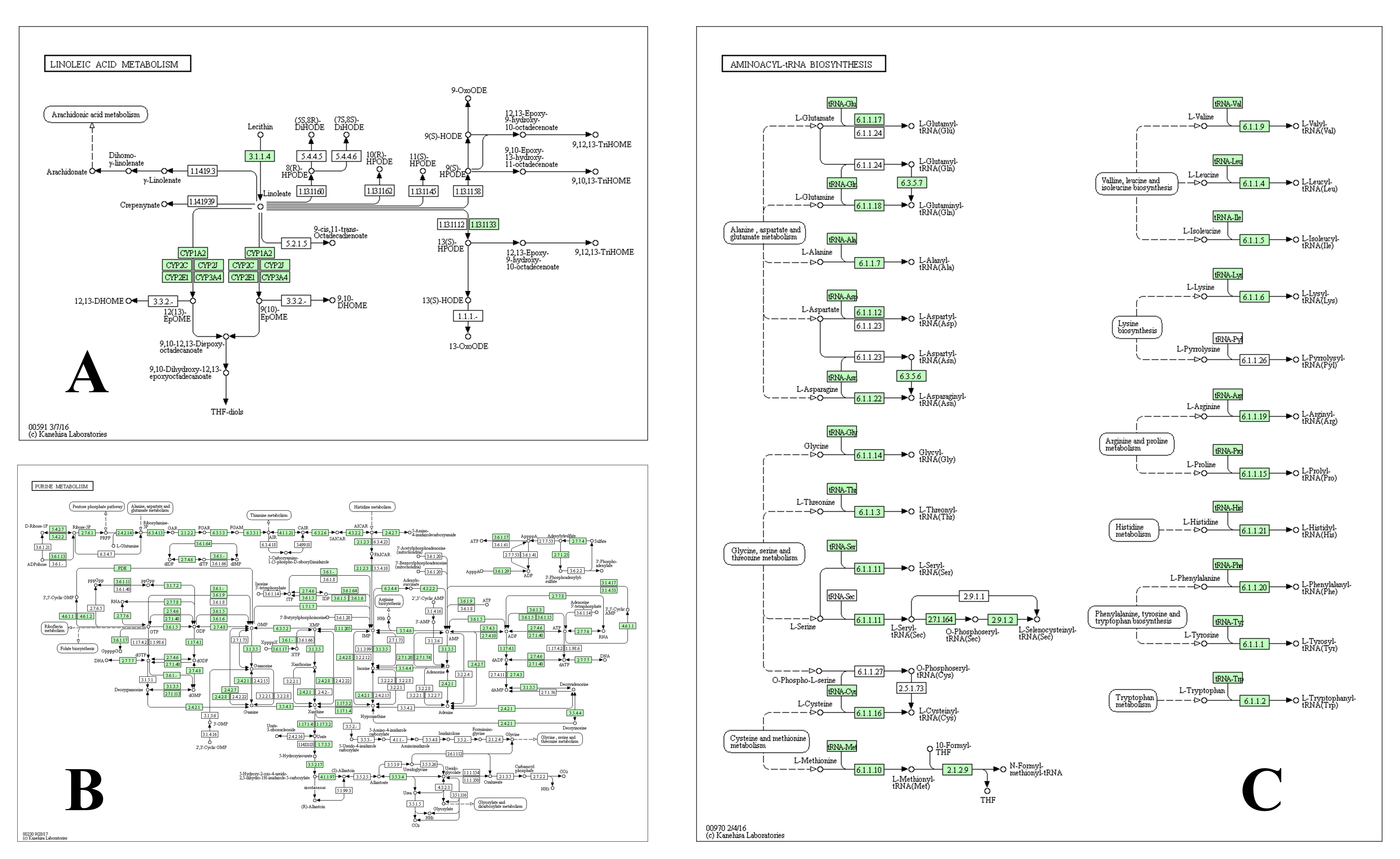


**Figure S16.** The pathway network in KEGG database of some important pathways included in pathway enrichment analysis. (A) Linoleic acid metabolism. (B) Purine metabolism. (C)Aminoacyl-tRNA biosynthesis[^1-3^](#_ENREF_1). (Permission of KEGG, Ref: 180019)

**Table S1.** Clinical observation

| No. | [Obvervational](../../../../D:/Program%20Files/Dict/7.2.0.0703/resultui/dict/%3Fkeyword=obvervational) [index](../../../../D:/Program%20Files/Dict/7.2.0.0703/resultui/dict/%3Fkeyword=index) | No. | [Obvervational](../../../../D:/Program%20Files/Dict/7.2.0.0703/resultui/dict/%3Fkeyword=obvervational) [index](../../../../D:/Program%20Files/Dict/7.2.0.0703/resultui/dict/%3Fkeyword=index) |
| --- | --- | --- | --- |
| 1 | [No adverse reaction](../../../../D:/Program%20Files/Dict/7.2.0.0703/resultui/dict/javascript:%3B) | 21 | [Lacrimation](../../../../D:/Program%20Files/Dict/7.2.0.0703/resultui/dict/%3Fkeyword=lacrimation) |
| 2 | Death | 22 | [Salivation](../../../../D:/Program%20Files/Dict/7.2.0.0703/resultui/dict/%3Fkeyword=salivation) |
| 3 | Skin redness | 23 | [Bradypnea](../../../../D:/Program%20Files/Dict/7.2.0.0703/resultui/dict/%3Fkeyword=bradypnea) |
| 4 | Slack skin | 24 | [Dyspnea](../../../../D:/Program%20Files/Dict/7.2.0.0703/resultui/dict/%3Fkeyword=dyspnea) |
| 5 | [Piloerection](../../../../D:/Program%20Files/Dict/7.2.0.0703/resultui/dict/%3Fkeyword=piloerection) | 25 | [Cheyne-Stokes](../../../../D:/Program%20Files/Dict/7.2.0.0703/resultui/dict/%3Fkeyword=Cheyne-Stokes) [breathing](../../../../D:/Program%20Files/Dict/7.2.0.0703/resultui/dict/%3Fkeyword=breathing) |
| 6 | Mucous mucus | 26 | [Lie](../../../../D:/Program%20Files/Dict/7.2.0.0703/resultui/dict/%3Fkeyword=lie) [down](../../../../D:/Program%20Files/Dict/7.2.0.0703/resultui/dict/%3Fkeyword=down) |
| 7 | Mucosa hyperemia | 27 | [Scream](../../../../D:/Program%20Files/Dict/7.2.0.0703/resultui/dict/%3Fkeyword=scream) |
| 8 | Hemolytic purple of the mucosa | 28 | [Fremitus](../../../../D:/Program%20Files/Dict/7.2.0.0703/resultui/dict/%3Fkeyword=fremitus) |
| 9 | Pale mucous membranes | 29 | [Ataxia](../../../../D:/Program%20Files/Dict/7.2.0.0703/resultui/dict/%3Fkeyword=ataxia) |
| 10 | Oral ulcer | 30 | [Convulsions](../../../../D:/Program%20Files/Dict/7.2.0.0703/resultui/dict/%3Fkeyword=convulsions) |
| 11 | [Ptosis of upper eyelid](../../../../D:/Program%20Files/Dict/7.2.0.0703/resultui/dict/javascript:%3B) | 31 | Direct movement |
| 12 | Exophthalmos | 32 | [Irritability](../../../../D:/Program%20Files/Dict/7.2.0.0703/resultui/dict/%3Fkeyword=irritability) |
| 13 | [Nystagmus](../../../../D:/Program%20Files/Dict/7.2.0.0703/resultui/dict/%3Fkeyword=nystagmus) | 33 | [Muscle](../../../../D:/Program%20Files/Dict/7.2.0.0703/resultui/dict/%3Fkeyword=muscle) [rigidity](../../../../D:/Program%20Files/Dict/7.2.0.0703/resultui/dict/%3Fkeyword=rigidity) |
| 14 | Eyes cloudy | 34 | Over grooming |
| 15 | Elevated skin temperature | 35 | Circle round and round |
| 16 | Decreased skin temperature | 36 | Self-injury |
| 17 | [Emaciation](../../../../D:/Program%20Files/Dict/7.2.0.0703/resultui/dict/%3Fkeyword=emaciation) | 37 | [Retropulsion](../../../../D:/Program%20Files/Dict/7.2.0.0703/resultui/dict/%3Fkeyword=retropulsion) |
| 18 | Feces pulpy | 38 | Less activity |
| 19 | Mydriasis | 39 | Others |
| 20 | [Myosis](../../../../D:/Program%20Files/Dict/7.2.0.0703/resultui/dict/%3Fkeyword=myosis) | / | / |

| **Table S2.**  Weight of testis tissue. | | | |
| --- | --- | --- | --- |
| Group | Number | Weight | Unit |
| Control： | 001 | 98.1 | mg |
|  | 002 | 109.5 | mg |
|  | 003 | 80 | mg |
|  | 004 | 86.2 | mg |
|  | 005 | 94.6 | mg |
|  | 006 | 93.9 | mg |
| D1 (0.0015 mg/kg)： | A01 | 89.8 | mg |
|  | A02 | 86.9 | mg |
|  | A03 | 99.4 | mg |
|  | A04 | 76.7 | mg |
|  | A05 | 92.8 | mg |
|  | A06 | 77.5 | mg |
| D2 (1.5 mg/kg)： | B01 | 87.9 | mg |
|  | B02 | 87.6 | mg |
|  | B03 | 89.4 | mg |
|  | B04 | 86.7 | mg |
|  | B05 | 85.4 | mg |
|  | B06 | 75.5 | mg |
| D3 (10 mg/kg) : | C01 | 88.1 | mg |
|  | C02 | 85.4 | mg |
|  | C03 | 72.6 | mg |
|  | C04 | 80.9 | mg |
|  | C05 | 73.3 | mg |
|  | C06 | 67.9 | mg |
| D4 (30 mg/kg): | D01 | 89.7 | mg |
|  | D02 | 84.8 | mg |
|  | D03 | 88.3 | mg |
|  | D04 | 92.9 | mg |
|  | D05 | 96.1 | mg |
|  | D06 | 116.5 | mg |

**Table S3.** PLS-DA permutation test intercepts in testis, serum, urine.

|  | | | |  |
| --- | --- | --- | --- | --- |
| Sample | Group | Mode | Intercepts | |
|  |  |  | R2 | Q2 |
| Testis | 0.0015 mg/kg | POS | (0.0, 0.642) | (0.0, -0.241) |
|  |  | NEG | (0.0, 0.221) | (0.0, -0.434) |
|  | 1.5 mg/kg | POS | (0.0, 0.608) | (0.0, -0.271) |
|  |  | NEG | (0.0, 0.747) | (0.0, -0.274) |
|  | 10 mg/kg | POS | (0.0, 0.677) | (0.0, -0.247) |
|  |  | NEG | (0.0, 0.558) | (0.0, -0.146) |
|  | 30 mg/kg | POS | (0.0, 0.487) | (0.0, 0.-243) |
|  |  | NEG | (0.0, 0.453) | (0.0, -0.167) |
|  | All | POS | (0.0, 0.687) | (0.0, -0.936) |
|  |  | NEG | (0.0, 0.221) | (0.0, -0.434) |
| Serum | 0.0015 mg/kg | POS | (0.0, 0.679) | (0.0, -0.152) |
|  |  | NEG | (0.0, 0.702) | (0.0, -0.224) |
|  | 1.5 mg/kg | POS | (0.0, 0.68) | (0.0, -0.18) |
|  |  | NEG | (0.0, 0.678) | (0.0, -0.0688) |
|  | 10 mg/kg | POS | (0.0, 0.616) | (0.0, -0.253) |
|  |  | NEG | (0.0, 0.72) | (0.0, -0.0805) |
|  | 30 mg/kg | POS | (0.0, 0.658) | (0.0, -0.23) |
|  |  | NEG | (0.0, 0.757) | (0.0, -0.12) |
|  | All | POS | (0.0, 0.223) | (0.0, -0.406) |
|  |  | NEG | (0.0, 0.431) | (0.0, -0.651) |
| Urine | 0.0015 mg/kg | POS | (0.0, 0.755) | (0.0, -0.0415) |
|  |  | NEG | (0.0, 0.315) | (0.0, -0.173) |
|  | 1.5 mg/kg | POS | (0.0, 0.797) | (0.0, -0.0579) |
|  |  | NEG | (0.0, 0.685) | (0.0, -0.163) |
|  | 10 mg/kg | POS | (0.0, 0.726) | (0.0, -0.0674) |
|  |  | NEG | (0.0, 0.558) | (0.0, -0.146) |
|  | 30 mg/kg | POS | (0.0, 0.76) | (0.0, -0.109) |
|  |  | NEG | (0.0, 0.928) | (0.0, -0.168) |
|  | All | POS | (0.0, 0.157) | (0.0, -0.171) |
|  |  | NEG | (0.0, 0.275) | (0.0, -0.286) |

Reference:

1 Kanehisa, M., Sato, Y., Kawashima, M., Furumichi, M. & Tanabe, M. KEGG as a reference resource for gene and protein annotation. *Nucleic acids research* **44**, D457-462, doi:10.1093/nar/gkv1070 (2016).

2 Kanehisa, M. & Goto, S. KEGG: kyoto encyclopedia of genes and genomes. *Nucleic acids research* **28**, 27-30 (2000).

3 Kanehisa, M., Furumichi, M., Tanabe, M., Sato, Y. & Morishima, K. KEGG: new perspectives on genomes, pathways, diseases and drugs. *Nucleic acids research* **45**, D353-d361, doi:10.1093/nar/gkw1092 (2017).
